# Proteomics of Primary Uveal Melanoma: Insights into Metastasis and Protein Biomarkers

**DOI:** 10.3390/cancers13143520

**Published:** 2021-07-14

**Authors:** Geeng-Fu Jang, Jack S. Crabb, Bo Hu, Belinda Willard, Helen Kalirai, Arun D. Singh, Sarah E. Coupland, John W. Crabb

**Affiliations:** 1 Cole Eye Institute, Cleveland Clinic, Cleveland, OH 44195, USA; jangg@ccf.org (G.-F.J.); crabbj1@ccf.org (J.S.C.); singha@ccf.org (A.D.S.); 2 Lerner Research Institute, Cleveland Clinic, Cleveland, OH 44195, USA; 3 Department of Quantitative Health Sciences, Lerner Research Institute, Cleveland Clinic, Cleveland, OH 44195, USA; hub@ccf.org; 4 Proteomics and Metabolomics Facility, Lerner Research Institute, Cleveland Clinic, Cleveland, OH 44195, USA; willarb@ccf.org; 5 Liverpool Ocular Oncology Research Centre, Department of Molecular and Clinical Cancer Medicine, University of Liverpool, William Henry Duncan Building, West Derby Street, Liverpool L7 8TX, UK; h.kalirai@liverpool.ac.uk (H.K.); s.e.coupland@liverpool.ac.uk (S.E.C.); 6 Cleveland Clinic Lerner College of Medicine of Case Western Reserve University, Cleveland, OH 44106, USA; 7 Liverpool Clinical Laboratories, Liverpool University Hospitals NHS Foundation Trust, Duncan Building, Daulby Street, Liverpool L69 3GA, UK

**Keywords:** bioinformatics, immune profiling, iTRAQ, metastasis, prediction modeling, quantitative proteomics, uveal melanoma

## Abstract

**Simple Summary:**

This study pursued the proteomic analysis of primary uveal melanoma (pUM) for insights into the mechanisms of metastasis and protein biomarkers. Liquid chromatography tandem mass spectrometry quantitative proteomic technology was used to analyze 53 metastasizing and 47 non-metastasizing pUM. The determined proteome of 3935 proteins was very similar between the metastasizing and non-metastasizing pUM, but included the identification of 402 differentially expressed (DE) proteins. Bioinformatic analyses suggest significant differences in the immune response between metastasizing and non-metastasizing pUM. Immune protein profiling results were consistent with transcriptomic studies, showing the immune-suppressive nature and low abundance of immune checkpoint regulators in pUM, and suggest CDH1, HLA-DPA1, and several DE immune kinases and phosphatases as potential targets for immune therapy checkpoint blockade. Prediction modeling of the proteomic data identified 32 proteins capable of predicting metastasizing versus non-metastasizing pUM with 93% discriminatory accuracy.

**Abstract:**

Uveal melanoma metastases are lethal and remain incurable. A quantitative proteomic analysis of 53 metastasizing and 47 non-metastasizing primary uveal melanoma (pUM) was pursued for insights into UM metastasis and protein biomarkers. The metastatic status of the pUM specimens was defined based on clinical data, survival histories, prognostic analyses, and liver histopathology. LC MS/MS iTRAQ technology, the Mascot search engine, and the UniProt human database were used to identify and quantify pUM proteins relative to the normal choroid excised from UM donor eyes. The determined proteomes of all 100 tumors were very similar, encompassing a total of 3935 pUM proteins. Proteins differentially expressed (DE) between metastasizing and non-metastasizing pUM (*n* = 402) were employed in bioinformatic analyses that predicted significant differences in the immune system between metastasizing and non-metastasizing pUM. The immune proteins (*n* = 778) identified in this study support the immune-suppressive nature and low abundance of immune checkpoint regulators in pUM, and suggest CDH1, HLA-DPA1, and several DE immune kinases and phosphatases as possible candidates for immune therapy checkpoint blockade. Prediction modeling identified 32 proteins capable of predicting metastasizing versus non-metastasizing pUM with 93% discriminatory accuracy, supporting the potential for protein-based prognostic methods for detecting UM metastasis.

## 1. Introduction

Uveal melanoma (UM), the most common primary malignancy of the eye in adults [1], is a relatively rare but aggressive cancer that progresses to fatal metastasis in about 50% of patients [2,3]. The median survival time for UM patients is about 9 months after the detection of metastasis [4]. Primary UM (pUM) originates predominantly in the capillary-rich uveal tract (i.e., the iris, ciliary body, and choroid), which facilitates metastasis through hematogenous dissemination of the tumor cells. UM metastases usually target the liver, but multiple metastases in other organs (e.g., lung and bone) also occur [5], with micrometastases capable of lying dormant and undetected for decades [6]. While immunotherapy has been beneficial for treating metastatic skin melanoma, it is well known that uveal and cutaneous melanomas differ in many ways [7,8], and no treatments currently exist to effectively treat metastatic UM [9].

The mechanisms of UM metastasis remain poorly understood but involve multiple gene mutations and tumor dormancy [10]. Predominant gene mutations associated with UM metastasis include *BAP1* [11], *GNAQ*, and *GNA11* [12,13]. Other mutations have been found in *PLCB4* [14], *CYSLTR2* [15], *SF3B1* [16], and *EIF1AX* [17]. In addition, chromosomal abnormalities, including loss on chromosomes 1p, 3, 6q, 8p, and 9p and gain on chromosomes 1q, 6p, and 8q [1,3,18], as well as disruption of epigenetic regulators [19], have been associated with UM metastasis. Recent transcriptomic studies have implicated immune suppression in the mechanisms of UM metastasis [20,21,22]. Current UM prognostic methods rely on cyto- or molecular-genetic [23,24,25] and gene expression analyses [26,27,28] of pUM biopsies [1,29,30]. An urgent need exists for improved prognostic methods [31], including an effective liquid assay for circulating pUM cells, which could facilitate earlier detection and treatments [32].

This study pursued quantitative proteomic analysis of pUM for insights into the mechanisms of UM metastasis and biomarkers for protein-based methods of UM prognosis. This is the largest proteomic study of UM to-date and involves the characterization of 53 metastasizing and 47 non-metastasizing pUM using LC MS/MS iTRAQ technology. Previous proteomic studies of pUM tissues from our laboratory [33] and others [34,35,36] have been limited by small sample sizes. Previous in vitro UM proteomic studies have characterized the secretome and proteome of primary UM cell lines, cultured choroid melanocytes, cultured liver metastases [37,38,39,40,41,42,43], and have identified cargo in extracellular vesicles from cultured pUM [44]. This study identifies a significant number of differentially expressed pUM proteins that provide bioinformatic insights into the differences between metastasizing and non-metastasizing pUM, and a foundation for protein-based assays for UM metastasis.

## 2. Results

### 2.1. Primary UM Tumor Samples

The tumor specimens (*n* = 100) used in this study were collected at the Cleveland Clinic, Cleveland, OH, USA, and at the Ocular Oncology Biobank, University of Liverpool, Liverpool, UK. The specimens were derived from 53 metastasizing and 47 non-metastasizing pUM; donors included 53 males and 47 females, with an average age of 63 years old. The metastatic status of the pUM was established by a combination of detailed clinical data, as well as survival and prognostic analyses including gene expression, multiplex ligation-dependent probe amplification, fluorescent in situ hybridization, and genome wide single nucleotide analysis. Histopathology examinations of liver biopsies or liver metastasis resection specimens confirmed the metastatic status of all metastasizing pUM. Detailed properties of each pUM specimen are described in Table S1, including demographic and clinical characteristics, prognostic analyses, metastasis and survival status, chromosome 3 status, cell type, histopathology, and associations with same-eye choroid control specimens.

### 2.2. Proteomic Characterization of the Choroid Controls 

**Figure 1:**
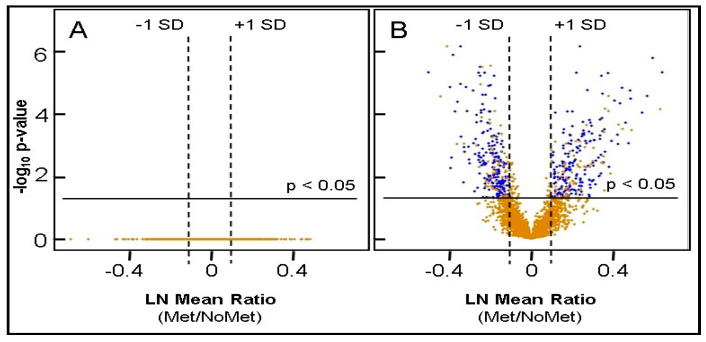
Volcano Plots. (**A**) Volcano plot for 2504 proteins from choroid specimens excised from 6 metastasizing and 7 non-metastasizing UM eyes (**B**) Volcano plot for 3935 proteins from 53 metastasizing pUM and 47 non-metastasizing pUM. Blue represents DE (differentially expressed) proteins and gold represents all other proteins not satisfying DE criteria. No significantly altered proteins were found in the choroid controls from UM eyes; 402 DE proteins were identified in the pUM.

The suitability of 13 choroid specimens from UM eyes to serve as control tissue was evaluated by histology and LC MS/MS iTRAQ analysis relative to the choroid pooled from nine disease-free eyes [33]. The results of these analyses are itemized in Table S2 and revealed no significant differences between the choroid specimens from metastasizing (*n* = 6) and non-metastasizing (*n* = 7) UM eyes, as illustrated by the flat distribution of protein ratios in the volcano plot in Figure 1A. The 13 choroid control tissues exhibited similar proteomes, consistent with the level of similarity observed in other normal tissues [45,46]. All 13 choroid specimens were accepted as controls based on (i) no significant differences between the specimens from metastasizing and non-metastasizing UM eyes and (ii) near-to-normal protein distribution (Figure S1). 

### 2.3. pUM Quantitative Proteomics Overview

As summarized in Table 1, a total of 3935 proteins were identified with two or more unique peptides using LC MS/MS and quantified by iTRAQ technology relative to a choroid control pooled from pUM-containing eyes. Very similar numbers of proteins were quantified from the metastasizing and non-metastasizing pUM, with 2555 proteins on average quantified per pUM specimen. The distribution of the protein ratios from metastasizing and non-metastasizing pUM was near-to-normal and statistically appropriate for comparative analyses (Figure S1). The quantitative results for each of the 100 pUM specimen are itemized in Table S3, including protein ratios, standard deviation (SD), and the total number of proteins quantified. The average relative abundance of the proteins quantified in the 53 metastasizing and 47 non-metastasizing pUM are presented in Tables S4 and S5, respectively. Significantly elevated or decreased proteins were defined as those exhibiting average protein ratios (pUM/control) above or below the mean by at least 1 SD with adjusted *p*-values ≤ 0.05 and containing ≤ 20% imputation of missing data. Significantly altered pUM proteins are highlighted by color coding in Tables S4 and S5 and illustrated by volcano plot in Figure 1B. The average determined proteomes of metastasizing and non-metastasizing pUM were very similar, with only about 11% of the total proteins significantly altered in abundance relative to the choroid control. As summarized in Table 1, metastasizing and non-metastasizing pUM exhibited similar numbers of significantly altered proteins, with each tumor group exhibiting slightly more elevated than decreased proteins. The significantly elevated proteins in metastasizing and non-metastasizing pUM differed in composition by about 65% (*n* = 148 proteins), while the significantly decreased proteins in the two tumor groups differed by about 25% (*n* = 52 proteins) in composition. Nevertheless, the proteomes of the metastasizing and non-metastasizing pUM appear to be about 89% similar over the 3935 identified proteins.

### 2.4. Independent Evidence Supporting the iTRAQ Protein Quantitation

Western blot analysis was used to independently evaluate the abundance of 12 proteins in 8 metastasizing pUM and 8 non-metastasizing pUM, relative to 8 normal choroid control tissues from pUM-containing eyes. Densitometric analysis of SDS-PAGE 

**Figure 2:**
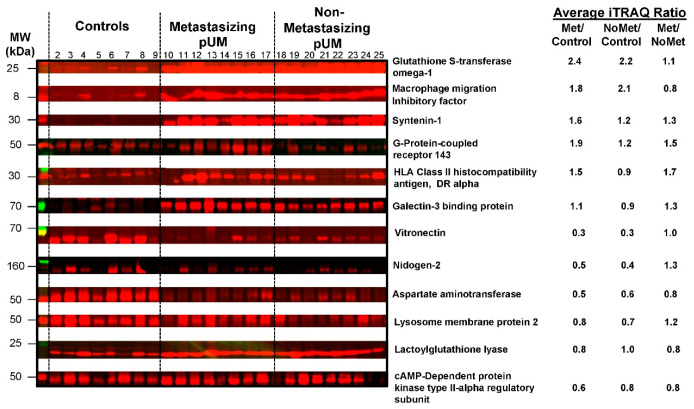
Western blot analysis. Fluorescence immunoblot reactivity is shown for 12 proteins quantified by LC MS/MS iTRAQ technology in choroid control tissues (lanes 2–9), metastasizing pUM (lanes 10–17), and non-metastasizing pUM (lanes 18–25). Prior to blotting, sample amounts applied to SDS-PAGE (~10 μg) were equalized based on Coomassie blue staining intensities (see Supplementary Figure S2). Western blot immunoreactivity (Supplementary Figure S3) supports the average iTRAQ protein ratios shown for metastasizing pUM (Met/control), non-metastasizing pUM (NoMet/control), and Met pUM/NoMet pUM.

Coomassie blue staining (Figure S2) was used to demonstrate the equal sample loading of all tissues prior to electroblotting to the PVDF membrane. Target protein immunoreactivity in each of the 12 Western blots (Figure S3) was quantified by densitometry and supported the iTRAQ protein quantitation. An overview of the immunoreactivity for each of the proteins is presented in Figure 2, along with the average iTRAQ ratios determined by LC MS/MS for 53 metastasizing and 43 non-metastasizing pUM.

### 2.5. Identification of Differentially Expressed Proteins

Differentially expressed (DE) proteins were sought through statistical comparison of the average protein ratios from metastasizing and non-metastasizing pUM. From the 3935 pUM proteins quantified, 583 proteins were identified with an adjusted *p*-value ≤ 0.05 for the average protein ratios (metastasizing pUM/non-metastasizing pUM), of which several exhibited low sample frequencies. From the 583 proteins, a total of 402 DE proteins were selected (Table S6) that contained no more than 20% imputed missing data and met the criteria of a minimum fold-change of one standard deviation (SD) from the mean, in addition to an adjusted *p*-value ≤ 0.05. Notably, 326 (81%) of the 402 DE proteins were detected in all 100 pUM with no missing data. Among the 402 DE proteins, 191 proteins were more abundant in metastasizing pUM and 211 proteins were more abundant in non-metastasizing pUM. Of potential utility in a future liquid assay for UM metastasis, 119 were predicted by gene ontology (GO) to be cell surface (plasma membrane) proteins. The most abundant and least abundant DE proteins in metastasizing pUM are shown in Table 2.

### 2.6. Bioinformatic Differences between Metastasizing and Non-Metastasizing pUM

**Figure 3:**
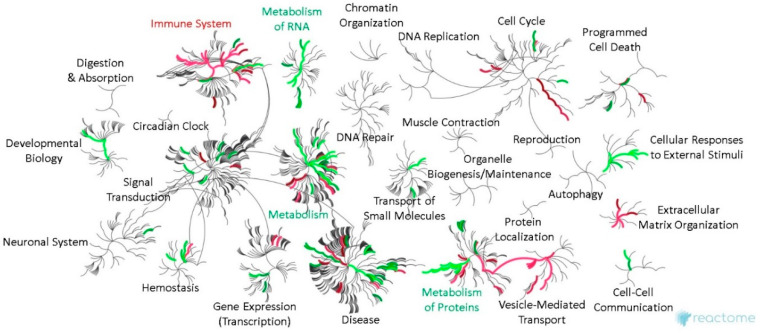
Genome-wide overview of bioinformatic pathways in metastasizing and non-metastasizing pUM. Reactome pathway analysis results are illustrated in network view for DE proteins elevated in metastasizing pUM (*n* = 191, red) and for those elevated in non-metastasizing pUM (*n* = 211, green). Top level Reactome pathways are labeled and displayed in circular bursts, with each step away from the center representing a lower level in pathway hierarchy. The color coding reflects over-representation of the pathway and no color signifies little, if any, pathway representation. See Supplementary Tables S7 and S8 for pathway details.

**Figure 4:**
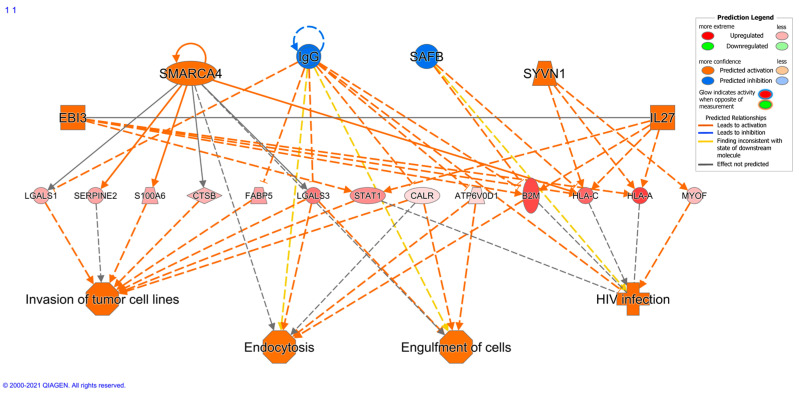
Regulatory effects network. The above upstream regulator effects network was predicted by the IPA bioinformatic analysis of the 191 DE proteins elevated in metastasizing pUM. Symbols: circle, complex/group; square—cytokine; vertical oval—transmembrane receptor; horizontal oval—transcription regulator; trapezoid—transporter. Solid lines represent direct interactions; dashed lines represent indirect interactions. IPA predicted no regulatory effects network for DE proteins elevated in non-metastasizing pUM.

Bioinformatic differences between metastasizing and non-metastasizing pUM were sought for insights into the mechanisms of UM metastasis. Reactome pathway analysis of the DE proteins elevated in metastasizing pUM (*n* = 191) predicted significant over-representation of immune system pathways, and, to a lesser extent, the pathways associated with vesicle-mediated trafficking, extracellular matrix organization, metabolism of proteins, and hemostasis. In contrast, the Reactome pathway analysis of the DE proteins elevated in non-metastasizing pUM (*n* = 211) predicted a significant over-representation of the pathways involving metabolism, including metabolism of proteins and RNA, and to a lesser extent, cellular response to external stimuli and developmental biology. A genome-wide overview illustrating these predicted pathway differences is shown in Figure 3, with pathway details provided in Tables S7 and S8 for DE proteins elevated in metastasizing and non-metastasizing pUM, respectively. Consistent with the Reactome analyses, the ingenuity pathway analysis (IPA) predicted the top functions for DE proteins elevated in metastasizing pUM to be associated with cellular compromise, molecular transport, cellular assembly and organization, cellular function and maintenance, and cell morphology. The top functions predicted by IPA for DE proteins elevated in non-metastasizing pUM involved protein synthesis, RNA damage and repair, RNA post-transcriptional modification, gene expression, and carbohydrate metabolism. IPA also predicted the regulator effects network, shown in Figure 4, from the 191 DE proteins elevated in metastasizing pUM. All 13 target genes shown in Figure 4 were detected in the proteomic analysis, as well as 4 of the 6 upstream regulators, namely *SMARCA4*, *IgG*, *SAFB*, and *SYNV1*. The target genes impact a number of cancer-related functions including invasion of tumor cells, endocytosis, and engulfment of cells.

### 2.7. Immune Protein Profiling

Recent transcriptomic investigations have reported that the tumor microenvironment (TME) in UM is immunosuppressive and contains relatively low amounts of conventional immune checkpoint regulators (ICRs) [20,21,22]. Toward the corroboration of these transcriptomic results and a better understanding of the UM immune response, we sought the identity of immune proteins within the determined pUM proteome. Our immune protein profiling corroborated the transcriptomic findings and resulted in the detection of 778 pUM immune proteins, including 15 ICRs, 27 immunosuppressive proteins, and 143 DE immune proteins (Table S9). Among the 143 DE immune proteins, 83 proteins were more abundant in metastasizing pUM and 60 proteins were more abundant in non-metastasizing pUM; all are tabulated with quantitation, frequency, and immune functional themes in Table S10. The detected ICRs (*CDH1*, *FYN*, *HLA-DPA1*, *HLA-DPB1*, *HLA-DQB1*, *HMGB1*, *LYN*, *PPP2CA*, *PPP2CB*, *PPP2R1A*, *PPP2R5A*, *PPP2R5C*, *PPP2R5E*, *PTPN11*, and *PTPN6*) were all of average to low abundance (Table S9), except for *CDH1* and *HLA-DPA1. CDH1* and *HLA-DPA1* were more abundant in metastasizing pUM than the choroid control (Table S9). Five DE immune proteins were among the 27 immunosuppressive proteins (Tables S9 and S10), including four elevated in metastasizing pUM (*HLA-DRA*, *LGALS3*, *STAT1*, and *TMED2*) and one (*PDHB*) more abundant in non-metastasizing pUM.

**Figure 5:**
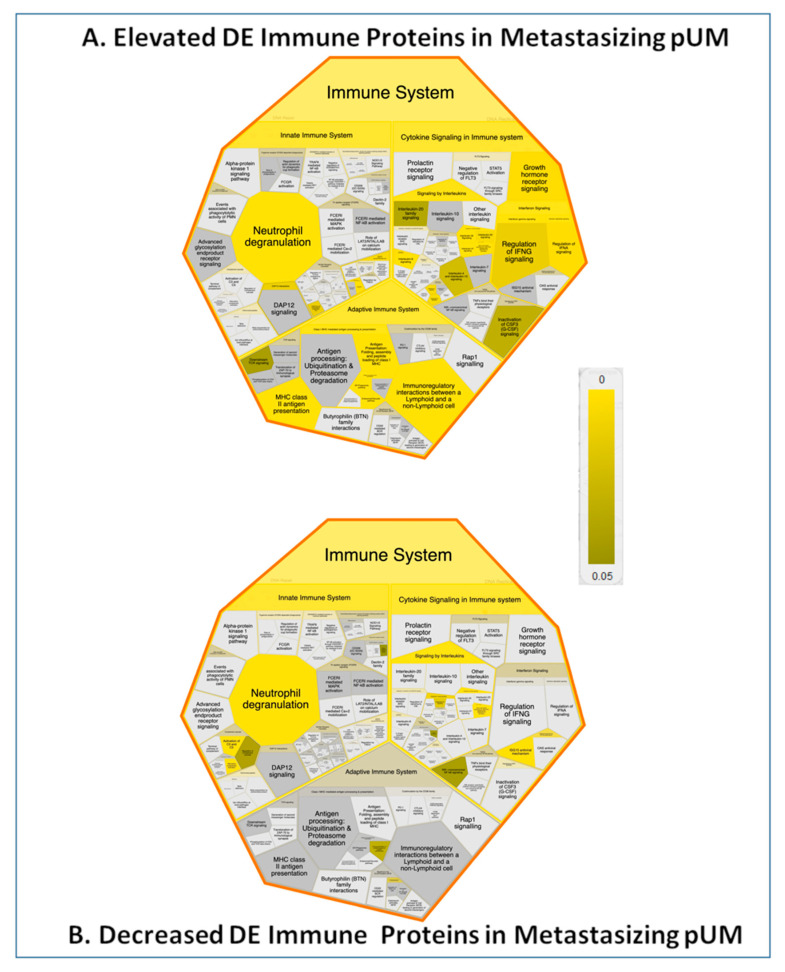
Immune System Pathways Associated with DE Immune Proteins. Reactome pathway analysis results are illustrated by Voronoi view for DE immune proteins elevated in metastasizing pUM (*n* = 83, Panel A) and for those elevated in non-metastasizing pUM (*n* = 60, panel B). Increased pathway over-representations are reflected by brighter colors. See Supplementary Tables S11 and S12 for details.

With the aim of better understanding the UM immune response, we pursued the identification of pathways associated with DE immune proteins. Reactome pathway analysis results for the 83 DE immune proteins elevated in metastasizing pUM and the 60 DE immune proteins elevated in non-metastasizing pUM are illustrated in Figure 5 and detailed in Tables S11 and S12, respectively. These results reinforce the major predicted difference between metastasizing and non-metastasizing pUM regarding immune system pathways, as addressed in the Discussion.

### 2.8. Prediction Modeling

**Figure 6:**
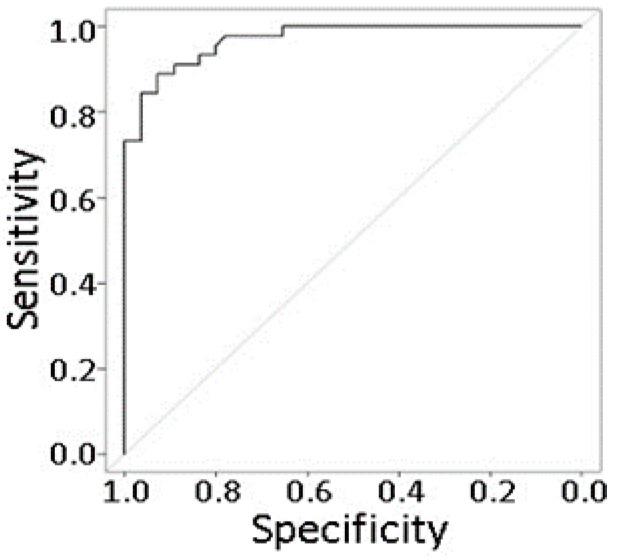
Receiver operating characteristic curve for the UM metastasis prediction model in Table 3. Corrected area under the ROC curve = 0.93.

Because improved UM prognostic methods are needed, we explored multiple statistical prediction models for UM metastasis using DE proteins with no missing data as predictors. Our final multivariate prediction model (Table 3) utilized 32 proteins selected by LASSO from 354 proteins with an adjusted *p*-value ≤ 0.05 and no missing data. In this model, 17 proteins were positively correlated with metastasis (i.e., elevated in metastasizing pUM), where eukaryotic translation initiation factor 4H had the strongest effect (OR = 2.02 per one unit increase in expression), followed by voltage-dependent anion-selection channel protein (OR = 1.73). Fifteen proteins were negatively correlated with metastasis (i.e., decreased in metastasizing pUM), where the odds ratios were 0.60 for Testis-expressed protein 10 and 0.63 for protein niban. Notably over 50% of the proteins in this model are predicted cell surface proteins (*n* = 18). The discriminatory accuracy of the model based on the corrected area under the ROC curve is 0.93 (Figure 6). At the optimal cut-off that maximizes the Youden index, the sensitivity of the model is 0.91 (95% CI = (0.79, 0.96)), and the specificity is 0.81 (95% CI = (0.66, 0.90)). These results support the feasibility of protein-based methods for a high accuracy detection of UM metastasis.

## 3. Discussion

In order to achieve a better understanding of the mechanisms of UM metastasis and protein biomarkers for UM metastasis, we pursued quantitative proteomics analysis of pUM using LC MS/MS iTRAQ technology. This is the largest quantitative proteomic study of this rare cancer to date and encompassed 100 pUM specimens, including 53 metastasizing and 47 non-metastasizing pUM. The specimens were collected at academic oncology centers in the UK and USA and all exhibited well-defined metastatic status from donor clinical records, health and survival histories, and genetic prognostic analyses. The status of all metastasizing specimens was confirmed by histopathology analysis of either liver biopsies or liver metastasis resection tissues. Metastatic deaths are most common in the first 10 years following UM diagnosis, with rare occurrences beyond 20 years [47]. Accordingly, it remains possible that some of the tumors classified as non-metastasizing in this study may become metastasizing melanoma over time, as proportions of the cured fraction evolve [48].

Quantitation of pUM protein was determined relative to pooled normal choroid tissue excised from six metastasizing and seven non-metastasizing pUM donor eyes, and each normal choroid specimen was validated by proteomic analysis to be a suitable choroid control component. A total of 3935 pUM proteins were quantified with at least two unique peptides, and the quantitation was independently supported by Western blot analysis. Overall, the average determined proteomes of the metastasizing and non-metastasizing pUM were very similar, with only about 11% of the total proteins exhibiting significant quantitative differences relative to the choroid control, as well as to each other. Based on rigorous statistical criteria, a total of 402 DE proteins were identified, including 191 DE proteins elevated in metastasizing pUM and 211 DE proteins elevated in non-metastasizing pUM. Our DE criteria includes a minimum fold change requirement of ± 1 SD from the mean, without which an additional 28 proteins could be classified as DE based on an adjusted *p*-value ≤ 0.05 and no missing data.

Although extensive gene expression analyses were not pursued, we did compare the 100 pUM proteomic dataset with transcriptomic results from The Cancer Genome Atlas (TCGA) study of 80 UM patients [49] for possible insights into the mechanisms of UM metastasis. The TCGA study divided UM patients into four biological subsets of metastasis risk (cluster one with the lowest risk to cluster four with the highest risk) based on genomic aberrations, transcriptional features, and clinical outcomes. The TCGA study also incorporated data from an independent gene expression study of 63 UM patients reported by Laurent et al. [50]. Table 4 provides a comparison of our proteomic data with TCGA and Laurent coding mRNA, including quantitative comparisons of transcripts grouped by somatic copy number alteration (SCNA) or by mRNA features in the two highest metastasis risk clusters, namely three and four. Table 4 shows that a majority (87–90%) of the 3935 proteins quantified in our study were detected in the TCGA and Laurent gene expression studies, including 91–94% of the DE proteins we identified, and with correlation levels of gene and protein expression (17–32%) consistent with literature values [51]. Table 4 also shows that TCGA and Laurent transcripts differentially abundant (DA) in clusters three versus four and grouped based on the somatic copy number alteration (SCNA) correlate well with 17 DE proteins more abundant and 2–3 DE proteins less abundant in metastasizing pUM. DE proteins associated with TCGA transcripts identified as up or down in cluster three versus cluster four and grouped based on mRNA correlate less well. Only ~28% of the DE proteins corresponding to transcripts up in cluster three were elevated in metastasizing pUM and only ~36% of the DE proteins corresponding to transcripts down in cluster three were decreased in metastasizing pUM. The DE proteins associated with these TCGA transcripts are provided in Table S13. Table 3 shows an excellent agreement between the DE proteins associated with TCGA and Laurent transcripts grouped by SCNA in clusters three versus four. Table 3 also shows the specific differences in DE proteins elevated or decreased in metastasizing pUM that are associated with the TCGA transcripts grouped by mRNA expression levels in clusters three versus four. These comparative analyses may be helpful for future studies, but also reinforce the value of coordinated protein and gene expression analyses of the same specimens.

In light of the significant proteomic similarities between metastasizing and non-metastasizing pUM, biological differences possibly contributing to metastasis were sought through bioinformatic analyses of 402 DE proteins. Two well-established bioinformatic analysis platforms (i.e., Reactome and IPA) suggested the most significant difference between metastasizing and non-metastasizing pUM was the over-representation of pathways in the immune system for proteins elevated in metastasizing pUM as opposed to proteins elevated in non-metastasizing pUM, which over-represented housekeeping pathways largely involving metabolism. DE proteins elevated in metastasizing pUM were predicted to function in processes involving the cytotoxicity of cells, stress response, disruption of the Golgi apparatus, degranulation (of neutrophils, lymphocytes, and platelets), transport, organization of organelles, endocytosis, homeostasis, and autophagy. In contrast, DE proteins elevated in non-metastasizing pUM were predicted to function in processes involving protein synthesis; translation of proteins; nonsense-mediated mRNA decay; and metabolism of RNA, proteins, and carbohydrates. IPA predicted an upstream regulator effects network for DE proteins elevated in metastasizing pUM that suggests hypotheses for testing regarding immune cell and tumor cell functions.

Bioinformatic analyses of 143 DE immune proteins identified in pUM support the notion that immune system pathways are strongly over-represented in metastasizing pUM, while housekeeping pathways are emphasized in non-metastasizing pUM. Reactome analysis of the 83 DE immune proteins elevated in metastasizing pUM (Table S11) provided several significant immune system pathway additions to those listed in Table S7, including MHC class II antigen presentation, *STING* mediated induction of host immune responses, *IRF3*-mediated induction of type I *IFN*, signaling by interleukins, gene and protein expression by *JAK*-*STAT* signaling after interleukin-12 stimulation, interleukin-35 signaling, and interleukin-6 signaling. Pathway analysis of the 60 DE immune proteins elevated in non-metastasizing pUM (Table S12) generated a few significant immune system pathways not listed in Table S8, including neutrophil degranulation, activation of C3 and C5, alternate complement activation, and signaling by interleukins. However, many other over-represented pathways were predicted for these 60 DE immune proteins, the most significant being associated with metabolism, signal transduction, hemostasis, and the transport of small molecules. Two limitations of our bioinformatic results warrant noting. First, the relatively small number of DE immune proteins (*n* = 143) available for analysis limited the pathways exhibiting both significant *p*-values and significant false discovery rates. Second, bioinformatic predictions generally evolve over time as the relevance of genes and proteins becomes better understood and the biological knowledgebase expands. Nevertheless, the bioinformatics results in this study suggest the immune system plays a significant role in metastasizing pUM. It will be important to localize the DE proteins within pUM and the TME to determine the degree of immune infiltration in metastasizing versus non-metastasizing pUM, and to facilitate the identification of therapeutic targets. This will be achievable, yet challenging, as only a fraction of the 402 DE proteins have so far been localized by immunohistochemical analyses. DE proteins localized to pUM cells include *HSPβ1* [52], *HLA-A* [53], *HLA-DRA* [53], *β2M* [53], *SDCBP* [54], *ATM* [55], and those localized to the TME include *LGALS3* [22] and *HLA-DRA* [56].

The apparent over-representation of immune system pathways in metastasizing pUM suggests an active immune response, despite metastatic UM patients being largely unresponsive to immunotherapy. Over-representation of immune system pathways in metastasizing pUM may be a compensatory mechanism to one or more malfunctioning immune components, as yet unknown. Evidence consistent with a compromised immune system can be found among the identified DE immune proteins (Table S10) and includes decreased amounts of key immune proteins in metastasizing pUM such as complement C3, complement factor B, and *CD81* antigen. Other DE immune proteins decreased in abundance in metastasizing pUM such as programmed cell death 6-interacting protein, cell death interacting protein 4, and heat shock protein beta-1 further suggest a compromised immune system in UM metastasis. Identifying key molecular weaknesses within the UM immune system remains a major challenge. 

**Figure 7:**
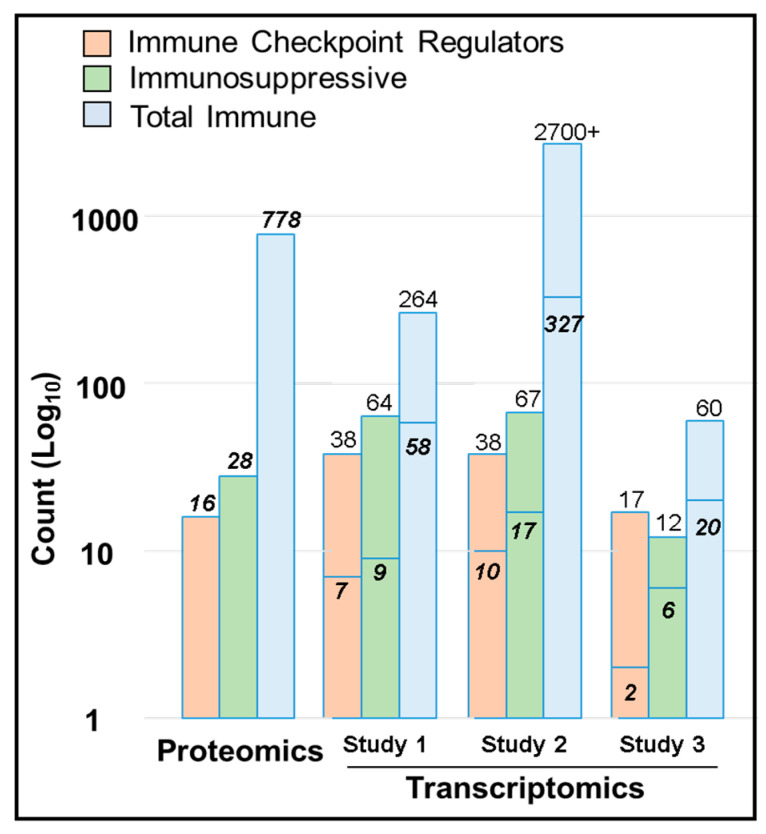
Immune Checkpoint Regulators and Immunosuppressive Proteins. The number of ICRs, immunosuppressive proteins, and total immune proteins quantified by proteomics in pUM is compared with similar immune transcripts reported in three recent transcriptomic studies. Transcriptomic Study 1 (Figueiredo et al. 2000) data is from pUM only; Transcriptomic Study 2 (Durante et al. 2000) includes data from both pUM and liver mUM; and Study 3 (Krishna et al. 2000) data is from liver mUM only.

UM patients show a limited response to immunotherapy, in contrast to patients with other cancers, such as cutaneous melanoma, where the immune checkpoint regulator (ICR) blockade has improved patient outcomes. Transcriptomic investigations have reported that conventional ICRs are in low abundance in pUMs [20,21], and our immunoprofiling results support this finding, despite the low correlation (~20%) between protein and gene expression in mammals [51]. Seven of the 16 ICRs we detected (Figure 7) were among the 38 ICRs also detected by Figueiredo et al. [21], including *CDH1*, *FYN*, *HLA-DPA1*, *HLA-DQB1*, *HMGB1*, *LYN*, and *NT5E*. Overall, we detected 22% of the 264 immune transcripts Figueiredo et al. [21] reported to be up or down regulated in the 80 pUM TCGA donor cohort [49]. Durante et al. [20] investigated both pUMs and liver metastases (mUMs) and reported over 2700 immune genes, of which we detected about 12% at the protein level (Figure 7). The Durante et al. study [20] reported a strong ICR gene expression of *LAG3*; variable expression of *TIGIT*; and minimal expression of *PDCD1*, *CTLA4*, *HAVCR2*, and *TNFRSF9*, none of which we detected at the protein level. We did detect 10 of the 38 ICRs in the Durante et al. [20] dataset (*FYN*, *HLA-DPA1*, *HLA-DPB1*, *HLA-DQB1*, *HMGB1*, *LYN*, *PPP2CA*, *PPP2R1A*, *PPP2R5C*, and *PTPN6*), all of which were of low to average abundance, except *HLA-DPA1*. Only two ICRs in our pUM proteomic dataset, namely *HMGB1* and *NT5E*, were among the 60 immune transcripts identified in the liver mUMs by Krishna et al. [22]. Overall, we detected 24 immune proteins in pUM in common with the liver mUM transcripts detected by Krishna et al. [22] and Figueiredo et al. [21].

Our proteomic results suggest two possible conventional ICR candidates for immune checkpoint blockades. We detected *CDH1* in all 100 specimens, and although not a DE protein, *CDH1* was significantly elevated in metastasizing pUM relative to the choroid control, and is an upregulated component of the widely used gene expression assay for UM metastasis [27]. *HLA-DPA1* was significantly more abundant in metastasizing pUM relative to both the choroid control and non-metastasizing pUM, and lacked DE status because it was detected in only 70% rather than 80% of the pUM. These results suggest *CDH1* and *HLA-DPA1* be considered as possible immunotherapy targets for blockade.

A final note is warranted regarding non-conventional ICRs. A majority of the 15 conventional ICRs (67%) detected in this study are enzymes functioning in phosphorylation and dephosphorylation, and include two kinases and eight phosphatases. Only two of these enzymes (*PPP2R1A* and *PTPN11*) were detected in all 100 pUMs and neither were significantly altered in abundance. Although not classified as ICRs, we detected 15 other kinases and phosphatases as DE immune proteins (Table S10). These enzymes include four kinases (*PDXK*, *ATM*, *PRKDC*, and *CSNK1A1*) and two phosphatases (*ACP2* and *PTPN1*) elevated in metastasizing pUM and seven kinases (*RPS6KA3*, *PGK1*, *PRKRA*, *RACK1*, *OXSR1*, *PPKAR2A*, and *PRHAR1A*) and two phosphatases (*PPP2R2A* and *PTPN23*) decreased in metastasizing pUM. Future investigations might consider evaluating the potential of these regulatory enzymes as ICRs in UM. More broadly, UM exhibits a high level of oxidative phosphorylation [57], and global phosphoproteomic studies are warranted to better understand the metabolic switches controlling pUM proliferation and to identify therapeutic targets.

Our proteomic results also support the recently reported immune-suppressive nature of UM tissues [20,21,22]. Ten of the 28 immunosuppressive proteins we detected in pUMs were among the 64 immunosuppressive genes reported by Figueiredo et al. [21], including four DE proteins (*HLA-A*, *HLA-DRA*, *LGALS3*, and *STAT1*). Seventeen of the 67 immunosuppressive genes identified in Durante et al. [20] were also detected at the protein level in this study, including five DE proteins (*HLA-A*, *HLA-DRA*, *LGALS3*, *STAT1*, and *TMED2*). Relative to liver mUM, the proteins we identified in pUMs were among the 23 immunosuppressive transcripts reported Krishna et al. [22] and Figueiredo et al. [21] in mUMs, including four DE proteins (*CD14*, *HLA-A*, *HLA-DRA*, and *LGALS3*).

Prediction modeling of the pUM proteomic dataset yielded 93% discriminatory accuracy in identifying metastasizing and non-metastasizing pUM, providing proof-of-concept that a high accuracy prediction of UM metastasis is possible based on protein expression. Current UM prognostic methods could and should be improved, as they poorly discriminate patients with the lowest metastatic risk from those with longer-term risk. Our proteomic results demonstrate that on a molecular level, protein-based UM prognostic methods would complement gene expression methods, as only 3 of the 12 genes used for the prognosis of UM metastasis based on gene expression [27] were detected in this study, namely *CDH1*, *FXR1*, and *LTA4H*. We used 32 pUM protein predictors in the current prediction model, but anticipate this number can be reduced further with additional research. While we used mass spectrometric technology to quantify proteins, the pUM protein expression could be measured rapidly with high sensitivity and specificity with a multiplex immunoassay. Our prediction modeling results provide a foundation for antibody selection for developing such an immunoassay, a technology with emerging potential in the analysis of UM serum and vitreous specimens [58,59]. About 56% of the predictors in our model are probable cell surface proteins, a property that can facilitate the development of a liquid assay for blood-borne pUMs. A liquid assay would provide a non-intrusive method for earlier detection of UM metastasis and monitoring of the disease progression and therapeutic efficacy. Our prediction modeling results, the continued detectability of pUM circulating tumor cells and DNA [32,60,61,62,63], and improved isolation methods [64] all provide support and encouragement for the future development of a liquid assay for UM metastasis.

## 4. Materials and Methods

### 4.1. Specimens

Primary uveal melanoma (pUM) samples were collected from UM patients undergoing ocular enucleation at the Cole Eye Institute, Cleveland Clinic (*n* = 37), and at the Department of Molecular and Clinical Cancer Medicine, University of Liverpool (*n* = 63). The UM eyes were from 53 males and 47 females and ranged in age from 28–86 years (average age of 63 years). Thirteen choroid specimens used in a pooled reference control for the proteomic analysis of the pUM were excised far from tumors in enucleated UM eyes collected at the University of Liverpool and are identified in Table S1. These choroid control specimens included six from metastasizing and seven from non-metastasizing pUM-containing eyes, of which eight were males and five were females, and exhibited an average donor age of about 65 years. Choroid tissues from nine disease-free postmortem eyes were obtained from the Cleveland Clinic Eye Bank, Cleveland, OH, and from the National Disease Research Interchange, Philadelphia, PA [33]; these tissues were used in a pooled control for proteomic analysis of the choroid excised from UM eyes. The metastatic status of the pUM specimens was established by a combination of detailed patient health histories and clinical survival data, and by fluorescent in situ hybridization analyses (FISH), genome-wide single-nucleotide polymorphism analysis (SNP), multiplex ligation-dependent probe amplification (MLPA), and/or gene expression analyses. Cytogenetic analyses for chromosomes 3 and 8 abnormalities by FISH were performed in the Department of Molecular Pathology, Cleveland Clinic; SNP analyses for chromosome 3 abnormalities were performed in the Genomics Core Facility at the Cleveland Clinic; and MLPA analyses for chromosomal deletions and duplications associated with UM were performed in the Department of Molecular and Clinical Cancer Medicine, University of Liverpool. The gene expression analyses for the 12 genes associated with the UM metastasis were performed at Castle Biosciences Inc., Phoenix, AZ. A histopathology analysis of the liver biopsies or liver metastasis resection specimens was employed to confirm the metastatic status of all of the metastasizing pUM. The properties of the pUM specimens used for the proteomic analysis are described in Table S1.

### 4.2. Sample Preparation

The pUM tissues (*n* = 100) and choroid control tissues from pUM-containing eyes (*n* = 13) were homogenized in 100 mM triethylammonium bicarbonate (TEAB) containing 2% SDS and 1 mM β-mercaptoethanol. Protein was extracted three times from the cell debris with centrifugation and the quantity of soluble protein was estimated by the bicinchoninic acid assay (Pierce) [65]. Each soluble protein fraction was reduced with 10 mM DTT, alkylated with 40 mM iodoacetamide, and then quenched with 40 mM DTT [66]. About 200 µg of reduced and alkylated protein from each fraction was precipitated with two volumes of ice-cold acetone. The protein pellets were resuspended in 100 mM TEAB buffer containing 0.5 mM CaCl_2_ and were digested overnight at 37 °C with trypsin (initially with 2% trypsin (*w*/*w*), followed in 2 h with another 2% (*w*/*w*), and the next day with another 1% (*w*/*w*) for 2h). Following proteolysis, soluble peptides were quantified by AccQ-Tag amino acid analysis [67,68]. Equal amounts of each of the 13 choroid specimens from the UM eyes were pooled to form a single reference control sample for the proteomic analysis of the 100 pUM. The preparation of the pooled choroid control from nine disease-free eyes was as previously described [33].

### 4.3. ITRAQ Labeling and Peptide Fractionation

iTRAQ labeling with an 8-plex iTRAQ kit was performed as previously described [33,68,69,70,71]. The choroid specimens from the UM eyes were first analyzed by LC MS/MS relative to the choroid from disease-free eyes. Tryptic digests of the 13 choroid specimens from UM eyes were each labeled with a single iTRAQ tag and combined in two unique batches with the pooled choroid control sample from nine disease-free eyes labeled with a unique iTRAQ tag. Specifically, one batch contained choroid specimens from four metastatic and three non-metastatic UM eyes (25 µg each), and the other batch contained choroid specimens from two metastatic and four non-metastatic UM eyes (25 µg each); both batches contained the disease-free choroid control (25 µg each). Each sample batch was individually fractionated by reverse-phase high performance liquid chromatography (RPHPLC) at pH 10 on a Waters xBridge BEH300 C18 column (3.5 µ particle size, 2.1 × 150 mm). Chromatography was performed at a flow rate of 200 µL/min using 0.1% NH_4_OH/aqueous acetonitrile solvents, a 0.7%/min acetonitrile gradient over 45 min; absorbance was monitored at 214 nm and the fractions were collected at 1 min intervals. Chromatography fractions encompassing the entire elution were selectively combined and dried, and a total of 12 fractions per batch were analyzed using LC MS/MS.

iTRAQ labeling of the pUM specimens proceeded after the 13 choroid tissues from the UM eyes were demonstrated by proteomic analysis to be suitable to serve in a pooled reference control. Tryptic digests of the 100 pUM (25 µg per sample) were each labeled with a single iTRAQ tag and combined in 15 unique batches (6–7 specimens per batch) with the pooled choroid control (25 µg/batch) that was also labeled with a unique iTRAQ tag. Each batch of six to seven specimens contained both metastasizing and non-metastasizing pUM and specimens from both Cleveland and Liverpool, whenever possible. Each batch of pUM specimens was fractionated by RPHPLC at pH 10, as described above, and the chromatography fractions were collected, combined, and dried for LC MS/MS analysis.

### 4.4. Protein Identification

RP-HPLC pH10 chromatography fractions were analyzed by LC MS/MS, as described elsewhere, using an Orbitrap Fusion Lumos Tribrid mass spectrometer [33,68,69,70,71]. Protein identification utilized the Mascot 2.6.2 search engine and the UniProt human reference proteome database version 2020_04 (20,376 human sequences). Database search parameters were restricted to three missed tryptic cleavage sites, a precursor ion mass tolerance of 10 ppm, a fragment ion mass tolerance of 20 mmu, and a false discovery rate of ≤1%. Protein identification required the detection of a minimum of two unique peptides per protein. Fixed protein modifications included N-terminal and ε-Lys iTRAQ modifications and S-carbamidomethyl-Cys. Variable protein modifications included Met oxidation, Asn and Gln deamidation, and iTRAQ Tyr. A minimum Mascot ion score of 25 was used for accepting the peptide MS/MS spectra.

### 4.5. Protein Quantitation

The iTRAQ tags on pUM peptides and choroid controls were quantified by the weighted average method [72] using the Mascot 2.6.2 Summed Intensities Program. Protein quantitation required a minimum of two unique peptides per protein, utilized a reporter ion tolerance of 10 ppm, and a Mascot peptide ion scores ≥ 25. Protein ratios were determined in log space and were transformed for reporting.

### 4.6. Statistical Analysis

Quantile normalization was used to normalize the mass spectrometry iTRAQ proteomics data. The missing protein expression data were further imputed using the *k*-nearest neighbor method. Batch effects were also examined. Means and standard error of the mean (SEM) were calculated for proteins quantified in metastasizing pUM (*n* = 53) and non-metastasizing pUM (*n* = 47). Differential expression (DE) analyses were performed using the limma package in R, and the results were adjusted for multiple-testing using the Benjamini–Hochberg procedure [73,74]. Criteria for DE proteins included average protein ratios (metastasizing pUM/non-metastasizing pUM) above or below the mean by at least one standard deviation, with adjusted *p*-values ≤ 0.05 and ≤20% for the imputed data. Further criteria for significantly elevated or decreased proteins included average ratios (pUM/control) above or below the mean by at least one standard deviation (SD), with adjusted *p*-values ≤ 0.05 and ≤20% for the imputed data. A minimum fold-change of 1 SD and a maximum of 20% allowance for missing data was incorporated into these criteria to minimize the impact of quantitative error on the identification of DE and significantly altered proteins.

A multivariate prediction model was pursued using pUM DE proteins as predictors. We explored three different modeling methods, namely logistic regression with the Least Absolute Shrinkage and Selection Operator (LASSO), logistic regression with the Akaike Information Criterion (AIC) to select predictors, and the Support Vector Machine (SVM). The model with the highest accuracy was chosen as the final model, for which we also constructed the receiver operating characteristics curve and evaluated the area under the curve with correction for optimism using Bootstrap. Sensitivity and specificity were computed to measure the model’s ability to discriminate between metastasizing and non-metastasizing pUM. All of the analyses were conducted with R 3.6.0 (cran.r-project.org, accessed date: 26 April 2021).

### 4.7. Bioinformatics

Bioinformatic analyses were performed with Ingenuity Pathways Analysis (IPA, Qiagen, Release Date 15 September 2020), NanoString Technologies, Seattle, WA (2019 and 2020 versions), the UniProt Knowledge Base (https://www.uniprot.org/, version August 2020, 25 March 2021), and the Reactome Pathway Browser [75] (https://reactome.org, version 76, 30 March 2021). Immune proteins within the determined pUM proteome were identified by interrogating a total of 2313 unique immune genes within gene panels from IPA and NanoString Technologies. Interrogated gene panels included 47 IPA immune response pathways containing 1515 unique genes and 2 NanoString Technologies panels (the nCounter PanCancer Immune Profiling Panel and the nCounter Immune Exhaustion Panel) representing 70 pathways and containing 1302 unique genes. In addition, immune proteins were sought among differentially expressed pUM proteins by gene ontology (GO) analysis using the UniProt Knowledge Base and Reactome Pathway Analysis. Categorization of immune suppressive and immune checkpoint regulator proteins was done as described by NanoString Technologies, Figueiredo et al. [21], Krishna et al. [22], and Waks et al. [76].

### 4.8. Western Blot Analysis

Western blot analysis [69,77] of the pUM and choroid control tissues was performed using 12% or 4–20% acrylamide Invitrogen/Novex precast SDS-PAGE gels (1 mm × 7 cm × 13 cm, ThermoFisher, Waltham, MA, USA), polyvinylidene fluoride (PVDF) membrane (Millipore Sigma, Burlington, MA, USA), and IRDye 680RD secondary antibody detection (LI-COR, Lincoln, NE, USA). Fluorescence was detected with a LI-COR Odyssey CLx imaging system with Image Studio 5.2. Prior to Western blot analysis, and the sample amounts applied to SDS-PAGE (~10 μg) were equalized based on Coomassie blue staining intensities [78] quantified by densitometry using a Bio-Rad GS-710 instrument and Bio-Rad Quantity One software 4.6.8. The PVDF membranes were blocked with a LI-COR Odessey blocking buffer and probed with primary antibodies at 4 °C overnight. The following 12 primary antibodies were utilized: anti-glutathione S-transferase Omega 1 (mouse monoclonal antibody (mAb) at 0.5 µg/mL, #MABN642, EMD Millipore); anti-macrophage migration inhibitory factor (mouse mAb at 1.6 µg/mL, #MAB289-100, R&D Systems); anti-syntenin-1 (rabbit pAb at 1:1000 dilution, #A5360, ABclonal, Woburn, MA, USA); anti-HLA class II histocompatibility antigen, DR alpha (rabbit pAb at 1:2000, #A11787, ABclonal); anti-galectin-3 binding protein (goat pAb at 0.2 µg/mL, #AF2226, R&D Systems, Minneapolis, MN, USA); anti-vitronectin (mouse mAb at 0.4 µg/mL, #MAB2349, R&D Systems); anti-nidogen-2 (goat pAb at 0.3 µg/mL, #AF3385, R&D Systems); anti-aspartate aminotransferase (rabbit pAb at 1:2000, #A6915, ABclonal); anti-lysosome membrane protein 2 (goat pAb at 0.2 µg/mL, #AF1966, R&D Systems); anti-guanine nucleotide-binding alpha-11 (rabbit pAb at 1:2000, #A2731, ABclonal); anti-lactoylglutathione lyase (rabbit pAb at 1:800, #A1932, ABclonal); and anti-cAMP-dependent protein kinase type II-alpha regulatory subunit (rabbit mAb at 0.6 µg/mL, # MAB8000, R&D Systems). Secondary antibodies were purchased from LI-COR, USA, and used at 1:5000 dilution, for 2–3 h at room temperature in the dark, and included the following: donkey anti-mouse IgG (#925-68072), donkey anti-goat IgG (#925-68074), and goat anti-rabbit IgG (#925-6807).

## 5. Conclusions

In conclusion, quantitative proteomic analysis of 100 pUM led to the identification of a significant number of differentially expressed proteins and insights into the bioinformatic differences between metastasizing and non-metastasizing pUM, including differences in the immune response. Immune profiling of the determined pUM proteome confirmed transcriptomic findings that the TME of UM is immune-suppressive and contains a low abundance of conventional immune check point regulators. The proteomic results suggest CDH1, HLA-DPA1, and several DE immune kinases and phosphatases as possible candidates for immune checkpoint blockade therapy. Prediction modeling of the proteomic data showed that metastasizing and non-metastasizing pUM can be identified with 93% discriminatory accuracy, supporting protein-based prognostic methods for detecting UM metastasis. Without effective treatments for metastatic UM, improved prognostic methods and earlier detection could enhance survival options.

## Figures and Tables

**Table 1:** Summary: pUM Quantitative Proteomic Results.

	Metastasizing	Non-Metastasizing
Total pUM specimens	53	47
Total Proteins Quantified with ≥2 peptides	3935	3934
Average number proteins quantified per tumor	2567	2541
Proteins Elevated ≥ 1SD from Mean, adjusted *p* ≤ 0.05, imputation ≤ 20%	232	224
Proteins Decreased ≥ 1SD from Mean, adjusted *p* ≤ 0.05, imputation ≤ 20%	206	201

**Table 2:** Differentially Expressed pUM Proteins.

UniProt Accession	Gene Name	Protein (Sorted by Decreasing Protein Ratio)	Protein Ratio Met/NoMet	Adjusted *p*-Value	Frequency	
					Metastasizing pUM	Non-Metastasizing pUM
***DE Proteins Most Abundant in Metastasizing pUM***						
P23381	WARS1	Tryptophan--tRNA ligase, cytoplasmic	1.906	4.6 × 10^−6^	53	47
P04439	HLA-A	HLA class I histocompatibility antigen, A alpha chain	1.818	1.6 × 10^−6^	53	47
P61769	B2M	Beta-2-microglobulin	1.768	2.7 × 10^−5^	47	39
P01903	HLA-DRA	HLA class II histocompatibility antigen, DR alpha chain	1.727	9.2 × 10^−5^	53	47
Q03518	TAP1	Antigen peptide transporter 1	1.630	3.2 × 10^−5^	53	47
P10321	HLA-C	HLA class I histocompatibility antigen, C alpha chain	1.624	1.8 × 10^−5^	52	46
Q8IVF2	AHNAK2	Protein AHNAK2	1.616	8.8 × 10^−4^	44	36
O95816	BAG2	BAG family molecular chaperone regulator 2	1.595	2.3 × 10^−7^	42	39
P07686	HEXB	Beta-hexosaminidase subunit beta	1.589	1.9 × 10^−4^	53	47
P33121	ACSL1	Long-chain-fatty-acid--CoA ligase 1	1.562	1.4 × 10^−5^	53	47
P17931	LGALS3	Galectin-3	1.549	4.6 × 10^−5^	52	47
P19971	TYMP	Thymidine phosphorylase	1.534	9.4 × 10^−4^	53	47
P51810	GPR143	G-protein coupled receptor 143	1.530	4.6 × 10^−4^	53	47
Q06210	GFPT1	Glutamine--fructose-6-phosphate aminotransferase [isomerizing] 1	1.505	3.8 × 10^−5^	53	47
Q9H3G5	CPVL	Probable serine carboxypeptidase CPVL	1.486	7.4 × 10^−4^	53	47
***DE Proteins Least Abundant in Metastasizing pUM***						
P04792	HSPB1	Heat shock protein beta-1	0.726	1.4 × 10^−5^	53	47
Q9UBI6	GNG12	Guanine nucleotide-binding protein G(I)/G(S)/G(O) subunit gamma-12	0.722	1.6 × 10^−3^	45	40
Q9BZQ8	NIBAN1	Protein Niban 1	0.720	1.2 × 10^−3^	53	47
Q8NC51	SERBP1	Plasminogen activator inhibitor 1 RNA-binding protein	0.718	1.6 × 10^−2^	53	47
P28161	GSTM2	Glutathione S-transferase Mu 2	0.710	5.4 × 10^−4^	53	47
Q9P0M6	MACROH2A2	Core histone macro-H2A.2	0.708	1.1 × 10^−5^	47	39
Q9NUJ1	ABHD10	Mycophenolic acid acyl-glucuronide esterase, mitochondrial	0.707	6.8 × 10^−7^	53	47
Q14240	EIF4A2	Eukaryotic initiation factor 4A-II	0.697	2.3 × 10^−7^	53	47
P05387	RPLP2	60S acidic ribosomal protein P2	0.697	4.1 × 10^−5^	53	47
P34913	EPHX2	Bifunctional epoxide hydrolase 2	0.688	8.4 × 10^−5^	53	47
Q02252	ALDH6A1	Methylmalonate-semialdehyde dehydrogenase [acylating], mitochondrial	0.681	1.3 × 10^−6^	53	47
P21266	GSTM3	Glutathione S-transferase Mu 3	0.676	2.6 × 10^−3^	53	47
P09211	GSTP1	Glutathione S-transferase P	0.673	5.5 × 10^−3^	53	47
Q02338	BDH1	D-beta-hydroxybutyrate dehydrogenase, mitochondrial	0.672	1.4 × 10^−5^	50	44
O75891	ALDH1L1	Cytosolic 10-formyltetrahydrofolate dehydrogenase	0.604	4.6 × 10^−6^	46	42

The above proteins were selected from 402 total differentially expresssed (DE) proteins identified by LC MS/MS iTRAQ technology (see Supplementary Table S6). Note that the protein ratio is expressed as metastasizing pUM (Met)/non-metastasizing pUM (noMet); DE proteins least abundant in metastasizing pUM are most abundant in non-metastasizing pUM.

**Table 3:** Selected Proteins in the Final Prediction Model for UM Metastasis.

Uniprot Accession	Gene Nmae	Protein	Regression Coefficients	Odds Ratio	Protein Ratio Met/NoMet	Cell Surface Localization
P04439	HLA-A	HLA class I histocompatibility antigen, A alpha chain	0.436	1.547	1.818	X
Q86UX7	FERMT3	Fermitin family homolog 3	0.028	1.029	1.419	
P04062	GBA	Lysosomal acid glucosylceramidase	0.499	1.647	1.412	
P67936	TPM4	Tropomyosin alpha-4 chain	0.061	1.063	1.330	
P21796	VDAC1	Voltage-dependent anion-selective channel protein 1	0.546	1.727	1.225	X
A0FGR8	ESYT2	Extended synaptotagmin-2	0.497	1.643	1.216	X
P13674	P4HA1	Prolyl 4-hydroxylase subunit alpha-1	−0.310	0.733	1.211	
P23368	ME2	NAD-dependent malic enzyme, mitochondrial	0.050	1.051	1.201	
Q15056	EIF4H	Eukaryotic translation initiation factor 4H	0.702	2.017	1.190	
P50570	DNM2	Dynamin-2	0.131	1.140	1.175	X
Q99829	CPNE1	Copine-1	0.072	1.075	1.174	X
Q9HD67	MYO10	Unconventional myosin-X	0.252	1.287	1.160	X
P49748	ACADVL	Very long-chain specific acyl-CoA dehydrogenase, mitochondrial	0.072	1.075	1.154	
Q00341	HDLBP	Vigilin	0.485	1.623	1.122	X
P48729	CSNK1A1	Casein kinase I isoform alpha	0.030	1.031	1.121	
P53621	COPA	Coatomer subunit alpha	0.490	1.632	1.116	
P11142	HSPA8	Heat shock cognate 71 kDa protein	0.018	1.018	1.096	X
P54920	NAPA	Alpha-soluble NSF attachment protein	0.050	1.051	1.095	X
Q13616	CUL1	Cullin-1	−0.178	0.837	0.903	X
Q9BPX5	ARPC5L	Actin-related protein 2/3 complex subunit 5-like protein	−0.102	0.903	0.895	
P38606	ATP6V1A	V-type proton ATPase catalytic subunit A	−0.124	0.883	0.885	X
Q9BR76	CORO1B	Coronin-1B	−0.110	0.896	0.874	X
Q96TA1	NIBAN2	Protein Niban 2	−0.448	0.639	0.864	X
Q14344	GNA13	Guanine nucleotide-binding protein subunit alpha-13	−0.213	0.808	0.844	X
P01024	C3	Complement C3	−0.124	0.883	0.840	X
Q8N1G4	LRRC47	Leucine-rich repeat-containing protein 47	−0.391	0.677	0.838	
Q14624	ITIH4	Inter-alpha-trypsin inhibitor heavy chain H4	−0.380	0.684	0.836	X
Q9NXF1	TEX10	Testis-expressed protein 10	−0.512	0.599	0.802	
P62899	RPL31	60S ribosomal protein L31	−0.094	0.911	0.795	
Q96I99	SUCLG2	Succinate--CoA ligase [GDP-forming] subunit beta, mitochondrial	−0.254	0.775	0.789	X
Q9BZQ8	NIBAN1	Protein Niban 1	−0.095	0.910	0.720	X
P28161	GSTM2	Glutathione S-transferase Mu 2	−0.171	0.843	0.710	

The above 32 protein prediction model for UM metastasis was generated with the LASSO modeling method and provided 93% discriminatory accuracy based on the AUC of the ROC curve in Figure 6. Cell surface localization reflects GO analysis prediction results for plasma membrane proteins.

**Table 4:** Comparison of UM Proteomic Results with TCGA and Laurent Gene Expression Data.

	TCGA Coding mRNA	Laurent Coding mRNA	TCGA Transcripts DA SCNA Cluster 3 vs. 4	TCGA + Laurent Transcripts DA SCNA Cluster 3 vs. 4	TCGA Transcripts Up in mRNA Cluster 3 vs. 4	TCGA Transcripts Down in mRNA Cluster 3 vs. 4
Number transcripts *	12,319	13,142	591	510	338	2172
Transcripts Identified at protein level *	3433	3524	133	128	109	373
Fraction of transcript detected as proteins	27.9%	26.8%	22.5%	25.1%	32.2%	17.2%
Total DE proteins	378	364	20	19	18	28
DE proteins Elevated in Mets	181	181	17	17	5	18
DE proteins Decreased in Mets	197	183	3	2	13	10

* From Robertson et al. 2017 Cancer Cell 32, 204.

## Data Availability

The original mass spectra are publicly available from MassIVE (http://massive.ucsd.edu, 17 May 2021) using the identifier MSV000087437.

## Supplementary Materials

Supplementary Tables are provided in Excel format, Read-Only. These tables do not require a password and the data is sortable. The following are available online at https://www.mdpi.com/article/10.3390/cancers13143520/s1, Figure S1: Protein distributions, Figure S2: SDS-PAGE for Western blots, Figure S3: Western Blots (*n* = 12), Table S1: Specimen Properties, Table S2: Choroid Control Individual LC MS/MS iTRAQ Data, Table S3: pUM Individual LC MS/MS iTRAQ Data, Table S4: Average Relative Protein Abundance, Metastasizing pUM, Table S5: Average Relative Protein Abundance, Non-Metastasizing pUM, Table S6: Differentially Expressed (DE) Proteins, Table S7: Pathways—DE Proteins Elevated in Metastasizing pUM, Table S8: Pathways—DE Proteins Elevated in Non-Metastasizing pUM, Table S9: Immune Proteins, Table S10: DE Immune Proteins, Table S11: Pathways—DE Immune Proteins Elevated in Metastasizing pUM, Table S12: Pathways—DE Immune Proteins Elevated in Non-Metastasizing pUM; Table S13: Comparison of DE Proteins and Proteins in the TCGA datasets.
