# Supplementary material for: Proteomics of Primary Uveal Melanoma: Insights into Metastasis and Protein Biomarkers
Source: Cancers (Basel). 2021 Jul 14;13(14):3520. doi: 10.3390/cancers13143520 (PMC8307952; doi:10.3390/cancers13143520)
Supplement: Supplementary file 1 [file cancers-13-03520-s001.zip › Figure S1-Protein Distributions.pdf]

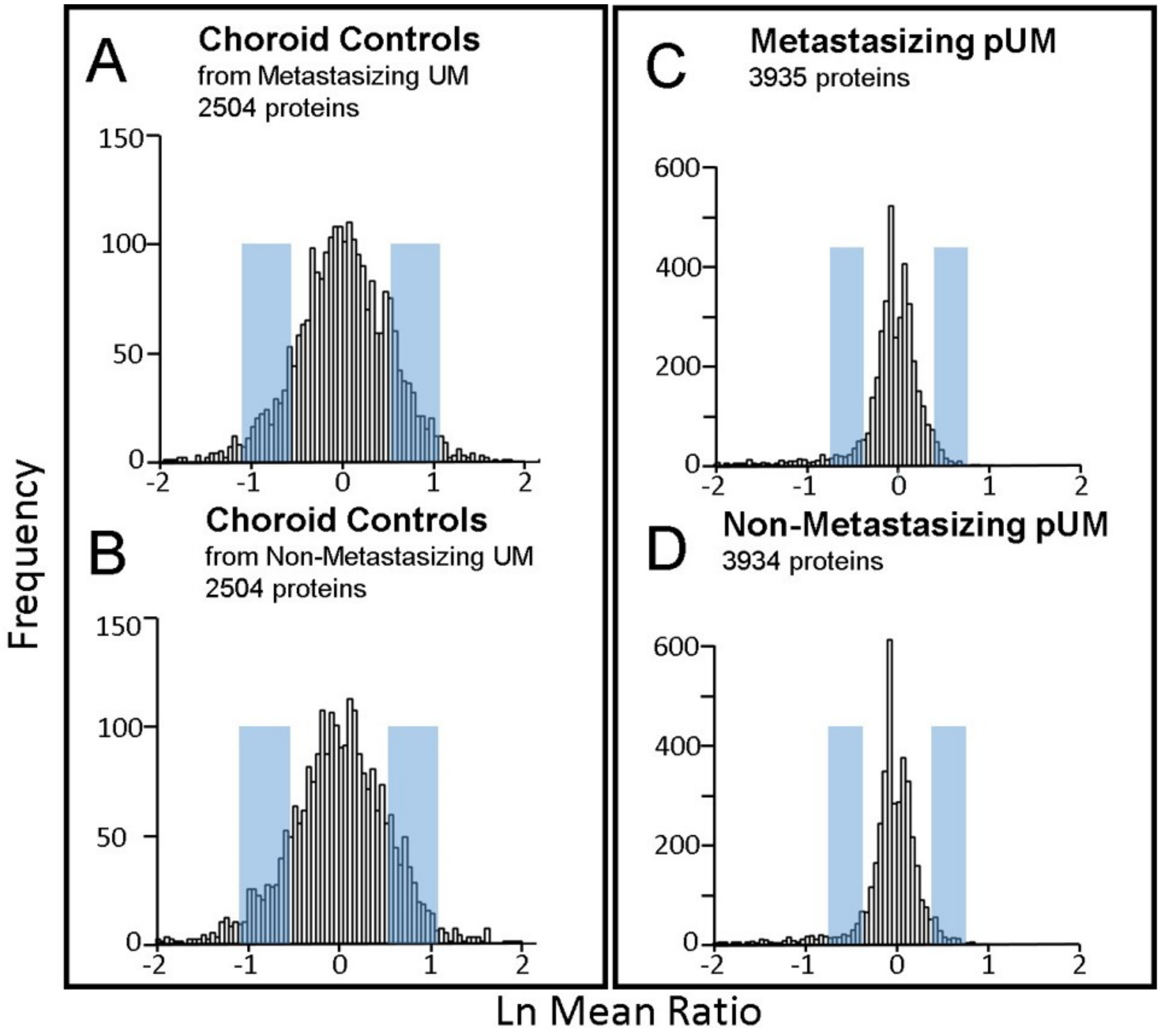

Supplementary Figure S1. Distribution of Protein Ratios. Determined LN mean protein ratios are shown for (A) choroid control from metastasizing UM eyes (n=6); (B) choroid control from non-metastasizing UM eyes (n=7); (C) metastasizing pUM (n=53); and (D) non-metastasizing pUM (n=47). Protein ratios between 1-2 SD from the mean are shaded. The histograms represent total proteins quantified with ≥ 2 unique peptides. The protein ratio distributions of all the groups are near-to-normal and statistically appropriate for comparative analyses.
