# Supplementary material for: Proteomics of Primary Uveal Melanoma: Insights into Metastasis and Protein Biomarkers
Source: Cancers (Basel). 2021 Jul 14;13(14):3520. doi: 10.3390/cancers13143520 (PMC8307952; doi:10.3390/cancers13143520)
Supplement: Supplementary file 1 [file cancers-13-03520-s001.zip › Figure S2-SDS-PAGE for Western blots.pdf]

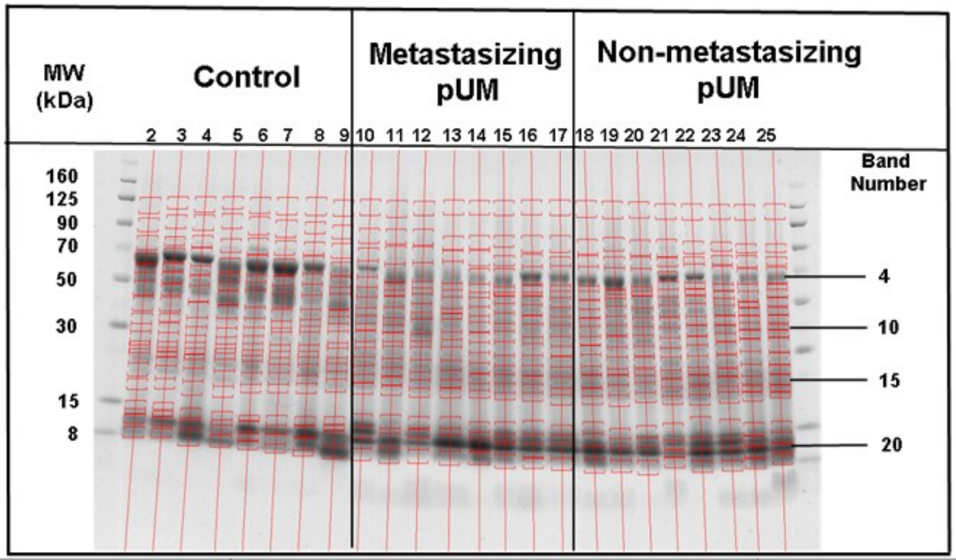


| Band Number | Control Density | | | | | | | | Metastasizing pUM Density | | | | | | | | Non-metastasizing pUM Density | | | | | | | |
|---|---|---|---|---|---|---|---|---|---|---|---|---|---|---|---|---|---|---|---|---|---|---|---|---|
| | Non-metastasizing | | | | Metastasizing | | | | | | | | | | | | | | | | | | | |
| Sample | C1 | C2 | C3 | C4 | C8 | C9 | C11 | C13 | UM91 | UM93 | UM94 | UM56 | UM62 | UM67 | UM85 | UM86 | UM34 | UM35 | UM39 | UM6 | UM9 | UM13 | UM17 | UM19 |
| Lane | 2 | 3 | 4 | 5 | 6 | 7 | 8 | 9 | 10 | 11 | 12 | 13 | 14 | 15 | 16 | 17 | 18 | 19 | 20 | 21 | 22 | 23 | 24 | 25 |
| 1 | 0.01 | 0.01 | 0.01 | 0.01 | 0.01 | 0.01 | 0.01 | 0.00 | 0.01 | 0.01 | 0.01 | 0.01 | 0.00 | 0.01 | 0.01 | 0.00 | 0.01 | 0.01 | 0.01 | 0.01 | 0.00 | 0.01 | 0.01 | 0.01 |
| 2 | 0.01 | 0.01 | 0.01 | 0.01 | 0.01 | 0.01 | 0.01 | 0.01 | 0.02 | 0.02 | 0.03 | 0.02 | 0.01 | 0.02 | 0.02 | 0.02 | 0.02 | 0.03 | 0.03 | 0.03 | 0.01 | 0.02 | 0.02 | 0.01 |
| 3 | 0.03 | 0.03 | 0.02 | 0.02 | 0.05 | 0.02 | 0.02 | 0.01 | 0.03 | 0.04 | 0.04 | 0.03 | 0.02 | 0.03 | 0.03 | 0.03 | 0.02 | 0.05 | 0.04 | 0.04 | 0.02 | 0.03 | 0.03 | 0.02 |
| 4 | 0.41 | 0.33 | 0.19 | 0.24 | 0.29 | 0.38 | 0.20 | 0.10 | 0.11 | 0.15 | 0.12 | 0.08 | 0.07 | 0.12 | 0.18 | 0.12 | 0.12 | 0.20 | 0.13 | 0.22 | 0.11 | 0.08 | 0.10 | 0.10 |
| 5 | 0.27 | 0.16 | 0.08 | 0.29 | 0.16 | 0.19 | 0.11 | 0.13 | 0.07 | 0.15 | 0.10 | 0.09 | 0.05 | 0.09 | 0.08 | 0.08 | 0.07 | 0.13 | 0.13 | 0.07 | 0.05 | 0.07 | 0.05 | 0.05 |
| 6 | 0.19 | 0.18 | 0.11 | 0.22 | 0.22 | 0.26 | 0.12 | 0.12 | 0.11 | 0.12 | 0.11 | 0.09 | 0.05 | 0.08 | 0.09 | 0.09 | 0.06 | 0.11 | 0.10 | 0.08 | 0.05 | 0.07 | 0.06 | 0.06 |
| 7 | 0.17 | 0.18 | 0.11 | 0.23 | 0.18 | 0.27 | 0.11 | 0.18 | 0.12 | 0.13 | 0.12 | 0.10 | 0.06 | 0.09 | 0.11 | 0.11 | 0.08 | 0.13 | 0.13 | 0.13 | 0.09 | 0.09 | 0.07 | 0.08 |
| 8 | 0.07 | 0.07 | 0.05 | 0.12 | 0.09 | 0.08 | 0.10 | 0.13 | 0.11 | 0.13 | 0.13 | 0.12 | 0.07 | 0.10 | 0.12 | 0.12 | 0.08 | 0.12 | 0.15 | 0.12 | 0.10 | 0.11 | 0.09 | 0.09 |
| 9 | 0.03 | 0.04 | 0.04 | 0.04 | 0.06 | 0.04 | 0.08 | 0.10 | 0.12 | 0.15 | 0.16 | 0.12 | 0.08 | 0.11 | 0.11 | 0.13 | 0.10 | 0.12 | 0.13 | 0.11 | 0.08 | 0.10 | 0.09 | 0.10 |
| 10 | 0.04 | 0.04 | 0.04 | 0.03 | 0.04 | 0.04 | 0.07 | 0.06 | 0.09 | 0.13 | 0.19 | 0.13 | 0.08 | 0.11 | 0.12 | 0.12 | 0.10 | 0.13 | 0.12 | 0.10 | 0.10 | 0.12 | 0.11 | 0.12 |
| 11 | 0.04 | 0.04 | 0.04 | 0.04 | 0.05 | 0.05 | 0.08 | 0.04 | 0.09 | 0.10 | 0.15 | 0.11 | 0.06 | 0.09 | 0.10 | 0.10 | 0.09 | 0.10 | 0.12 | 0.08 | 0.08 | 0.09 | 0.09 | 0.11 |
| 12 | 0.05 | 0.05 | 0.05 | 0.04 | 0.06 | 0.05 | 0.08 | 0.05 | 0.08 | 0.09 | 0.11 | 0.10 | 0.06 | 0.09 | 0.09 | 0.08 | 0.08 | 0.09 | 0.08 | 0.08 | 0.09 | 0.09 | 0.08 | 0.08 |
| 13 | 0.06 | 0.07 | 0.08 | 0.05 | 0.07 | 0.06 | 0.09 | 0.06 | 0.11 | 0.11 | 0.11 | 0.11 | 0.08 | 0.11 | 0.10 | 0.11 | 0.09 | 0.09 | 0.08 | 0.10 | 0.10 | 0.11 | 0.09 | 0.09 |
| 14 | 0.07 | 0.08 | 0.09 | 0.07 | 0.10 | 0.08 | 0.12 | 0.09 | 0.13 | 0.14 | 0.14 | 0.17 | 0.12 | 0.15 | 0.14 | 0.14 | 0.13 | 0.12 | 0.11 | 0.13 | 0.14 | 0.15 | 0.13 | 0.14 |
| 15 | 0.10 | 0.08 | 0.11 | 0.09 | 0.13 | 0.09 | 0.15 | 0.11 | 0.14 | 0.15 | 0.14 | 0.18 | 0.14 | 0.16 | 0.16 | 0.15 | 0.16 | 0.13 | 0.13 | 0.13 | 0.18 | 0.17 | 0.16 | 0.16 |
| 16 | 0.06 | 0.06 | 0.08 | 0.05 | 0.08 | 0.08 | 0.11 | 0.10 | 0.12 | 0.12 | 0.12 | 0.16 | 0.12 | 0.13 | 0.13 | 0.12 | 0.14 | 0.12 | 0.12 | 0.14 | 0.17 | 0.14 | 0.14 | 0.17 |
| 17 | 0.03 | 0.03 | 0.06 | 0.03 | 0.03 | 0.03 | 0.06 | 0.08 | 0.08 | 0.07 | 0.07 | 0.10 | 0.09 | 0.09 | 0.09 | 0.08 | 0.08 | 0.06 | 0.08 | 0.09 | 0.12 | 0.11 | 0.10 | 0.13 |
| 18 | 0.06 | 0.04 | 0.17 | 0.07 | 0.09 | 0.07 | 0.13 | 0.08 | 0.19 | 0.12 | 0.11 | 0.13 | 0.14 | 0.16 | 0.13 | 0.15 | 0.12 | 0.10 | 0.10 | 0.12 | 0.15 | 0.17 | 0.15 | 0.15 |
| 19 | 0.10 | 0.09 | 0.26 | 0.12 | 0.30 | 0.25 | 0.38 | 0.15 | 0.34 | 0.18 | 0.25 | 0.24 | 0.28 | 0.33 | 0.30 | 0.29 | 0.27 | 0.23 | 0.26 | 0.24 | 0.39 | 0.37 | 0.32 | 0.27 |
| 20 | 0.20 | 0.28 | 0.37 | 0.20 | 0.22 | 0.18 | 0.31 | 0.34 | 0.30 | 0.33 | 0.24 | 0.43 | 0.50 | 0.40 | 0.32 | 0.33 | 0.42 | 0.31 | 0.31 | 0.24 | 0.40 | 0.34 | 0.39 | 0.39 |
| 21 | 0.15 | 0.17 | 0.29 | 0.21 | 0.13 | 0.12 | 0.09 | 0.38 | 0.09 | 0.21 | 0.05 | 0.13 | 0.25 | 0.13 | 0.08 | 0.07 | 0.16 | 0.09 | 0.06 | 0.09 | 0.15 | 0.10 | 0.17 | 0.21 |
| Total Density | 2.13 | 2.01 | 2.26 | 2.17 | 2.37 | 2.34 | 2.39 | 2.33 | 2.46 | 2.65 | 2.51 | 2.64 | 2.35 | 2.60 | 2.49 | 2.43 | 2.41 | 2.45 | 2.42 | 2.32 | 2.59 | 2.53 | 2.44 | 2.54 |
| Average Density | 2.25 | | | | | | | | 2.52 | | | | | | | | 2.47 | | | | | | | |
| SD | 0.13 | | | | | | | | 0.11 | | | | | | | | 0.09 | | | | | | | |
| RSD (%) | 5.9 | | | | | | | | 4.5 | | | | | | | | 3.6 | | | | | | | |

**Supplementary Figure S2. SDS-PAGE for Western Blot Analysis.** Each lane in the above Coomassie blue stained SDS-PAGE contains the same tissue samples and protein amounts (~10 μg/lane) used for Western Blot Analysis to corroborate protein quantitation. The displayed densitometric data from Coomassie blue staining intensities supports equal sample loading per lane. Coomassie blue staining also demonstrates different SDS-PAGE profiles for pUM and control samples.
