# Supplementary material for: Proteomics of Primary Uveal Melanoma: Insights into Metastasis and Protein Biomarkers
Source: Cancers (Basel). 2021 Jul 14;13(14):3520. doi: 10.3390/cancers13143520 (PMC8307952; doi:10.3390/cancers13143520)
Supplement: Supplementary file 1 [file cancers-13-03520-s001.zip › Figure S3. Western Blots (n=12).pdf]

# *Supplementary Figure S3*

- ***Western blot results for 12 proteins.*** Each of the following Western blots in Figures S3,A-N include choroid control samples C01, C02, C03, C04, C08, C09, C11, C13, metastasizing pUM samples UM91, UM93, UM94, UM56, UM62, UM67, UM85, UM86, and non-metastasizing pUM samples UM33, UM34, UM39, UM06, UM09, UM13, UM17, UM19. Blue control sample labels denote choroid tissue from eyes containing metastazising pUM; green labels denote choroid from non-metastasizing pUM. Red pUM sample labels denote specimens from Cleveland, OH; black labels denote samples from Liverpool, UK. Prior to blotting to PVDF membrane, sample amounts applied to SDS-PAGE (~10 µg) were equalized based on Coomassie blue staining intensities (see Supplementary Figure S2). The Chameleon Duo pre-stained protein ladder from LI-COR, USA was used to estimate molecular weight. Fluorescence intensity was measured with a LI-COR Odyssey CLx imaging system on the indicated bands using Image Studio 5.2. Intensity measurements and calculations are presented without adjustments for background or outlier values.

***Figure S3A. Fluorescence immunoblot reactivity for glutathione S-transferase Omega-1, UniProt accession number P78471, molecular weight 27,566 Da.*** The blot was probed overnight at 4°C with anti-glutathione S-transferase Omega 1 (mouse mAb) at 0.5 µg/mL, #MABN642, EMD Millipore). Secondary antibody was donkey anti-mouse from LI-COR, USA (#925-68072) used at a dilution of 1:5000, 2-3 h, room temperature in the dark.

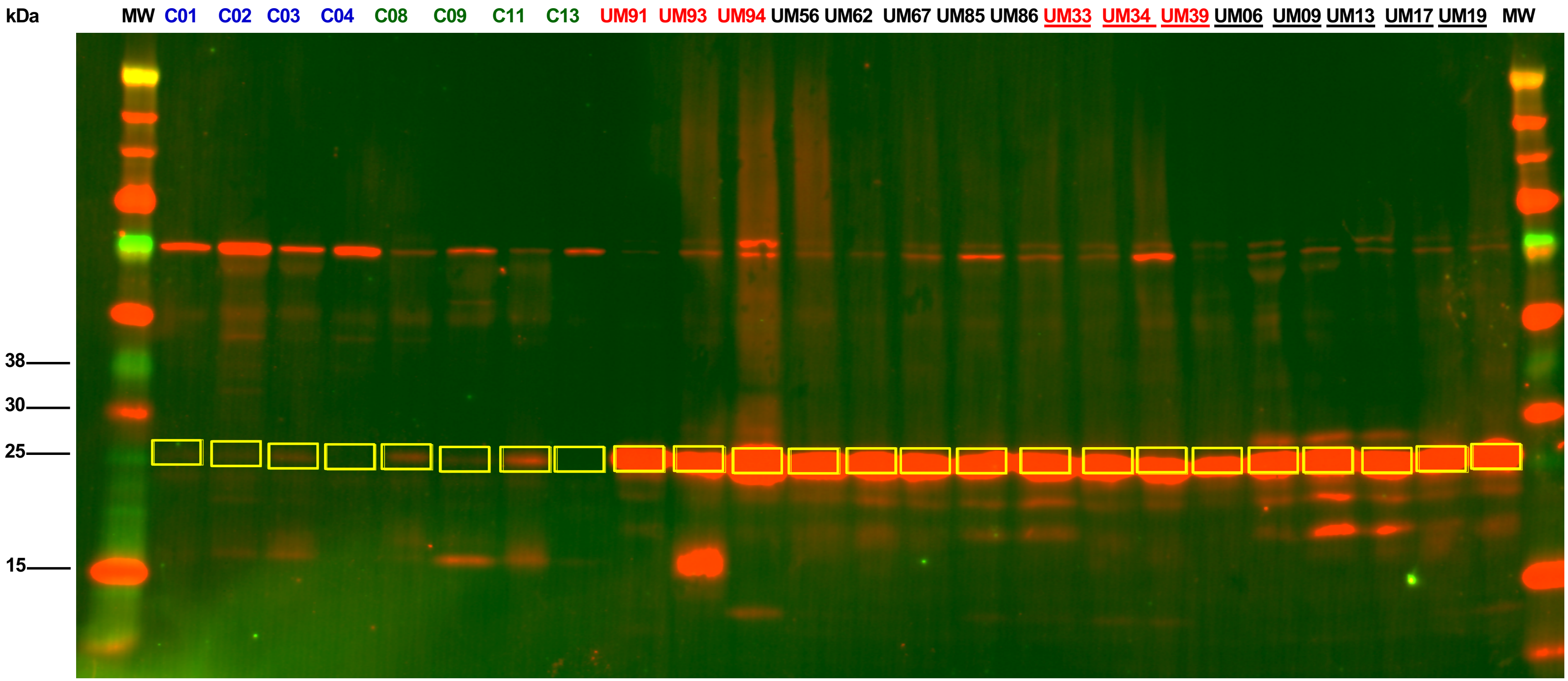


| Figure S3A. Quantitation | | | | | | | | | | | | | | | | | | | | | | | | |
|---|---|---|---|---|---|---|---|---|---|---|---|---|---|---|---|---|---|---|---|---|---|---|---|---|
| Glutathione S-tansferase Omega-1 | C01 | C02 | C03 | C04 | C08 | C09 | C11 | C13 | UM91 | UM93 | UM94 | UM56 | UM62 | UM67 | UM85 | UM86 | UM33 | UM34 | UM39 | UM06 | UM09 | UM13 | UM17 | UM19 |
| Fluorescence intensity | 1318 | 1357 | 1393 | 1224 | 1421 | 1287 | 1764 | 1133 | 20034 | 8406 | 30562 | 14072 | 14866 | 16734 | 17964 | 25003 | 15606 | 13011 | 8252 | 31118 | 32130 | 27001 | 17171 | 19923 |
| Average intensity ± RSD | 1362 ± 14% | | | | | | | | 18455 ± 37% | | | | | | | | 20526 ± 42% | | | | | | | |
| Average pUM intensity / Control ± RSD | | | | | | | | | 13.6 ± 37% iTRAQ=2.4 | | | | | | | | 15.1 ± 42% iTRAQ=2.2 | | | | | | | |
| Ave pUM Met / Ave pUM NoMet | | | | | | | | | 0.9 iTRAQ=1.1 | | | | | | | | | | | | | | | |

***Figure S3B. Fluorescence immunoblot reactivity for macrophage migration inhibitory factor, UniProt accession number P14174, molecular weight 12,476 Da.*** The blot was probed overnight at 4°C with anti-macrophage migration inhibitory factor (mouse mAb at 1.6 µg/mL, #MAB289-100, R&D Systems). Secondary antibody was donkey anti-mouse from LI-COR, USA (#925-68072) used at a dilution of 1:5000, 2-3 h, room temperature in the dark.

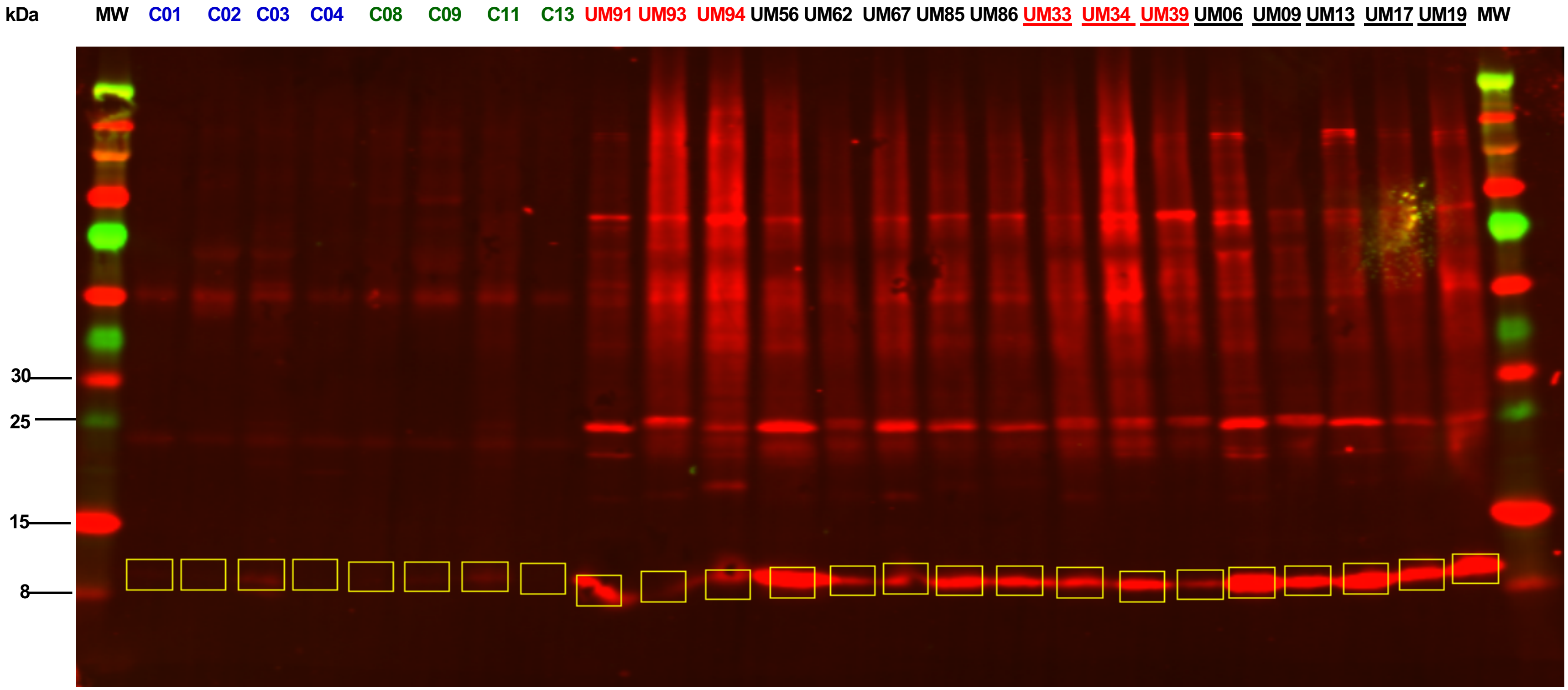


| Figure S3B. Quantitation | | | | | | | | | | | | | | | | | | | | | | | | |
|---|---|---|---|---|---|---|---|---|---|---|---|---|---|---|---|---|---|---|---|---|---|---|---|---|
| Macrophage migration inhibitory factor | C01 | C02 | C03 | C04 | C08 | C09 | C11 | C13 | UM91 | UM93 | UM94 | UM56 | UM62 | UM67 | UM85 | UM86 | UM33 | UM34 | UM39 | UM06 | UM09 | UM13 | UM17 | UM19 |
| Fluorescence intensity | 490 | 487 | 563 | 478 | 491 | 504 | 571 | 473 | 1116 | 543 | 731 | 2206 | 720 | 755 | 1049 | 911 | 851 | 1061 | 619 | 4202 | 1310 | 2697 | 1359 | 1996 |
| Average intensity ± RSD | 507 ± 8% | | | | | | | | 1004 ± 52% | | | | | | | | 1762 ± 67% | | | | | | | |
| Average pUM intensity / Control ± RSD | | | | | | | | | 2.0 ± 52% iTRAQ=1.8 | | | | | | | | 3.5 ± 67% iTRAQ=2.1 | | | | | | | |
| Ave pUM Met / Ave pUM NoMet | | | | | | | | | 0.6 iTRAQ=0.8 | | | | | | | | | | | | | | | |

***Figure S3C. Fluorescence immunoblot reactivity for Syntenin-1, UniProt accession number O00560, molecular weight 32,424 Da.*** The blot was probed overnight at 4°C with anti-syntenin-1 (rabbit pAb at 1:1000 dilution, #A5360, AB clonal). Secondary antibody was goat anti-rabbit IgG from LI-COR, USA (#925-6807) used at a dilution of 1:5000, 2-3 h, room temperature in the dark.

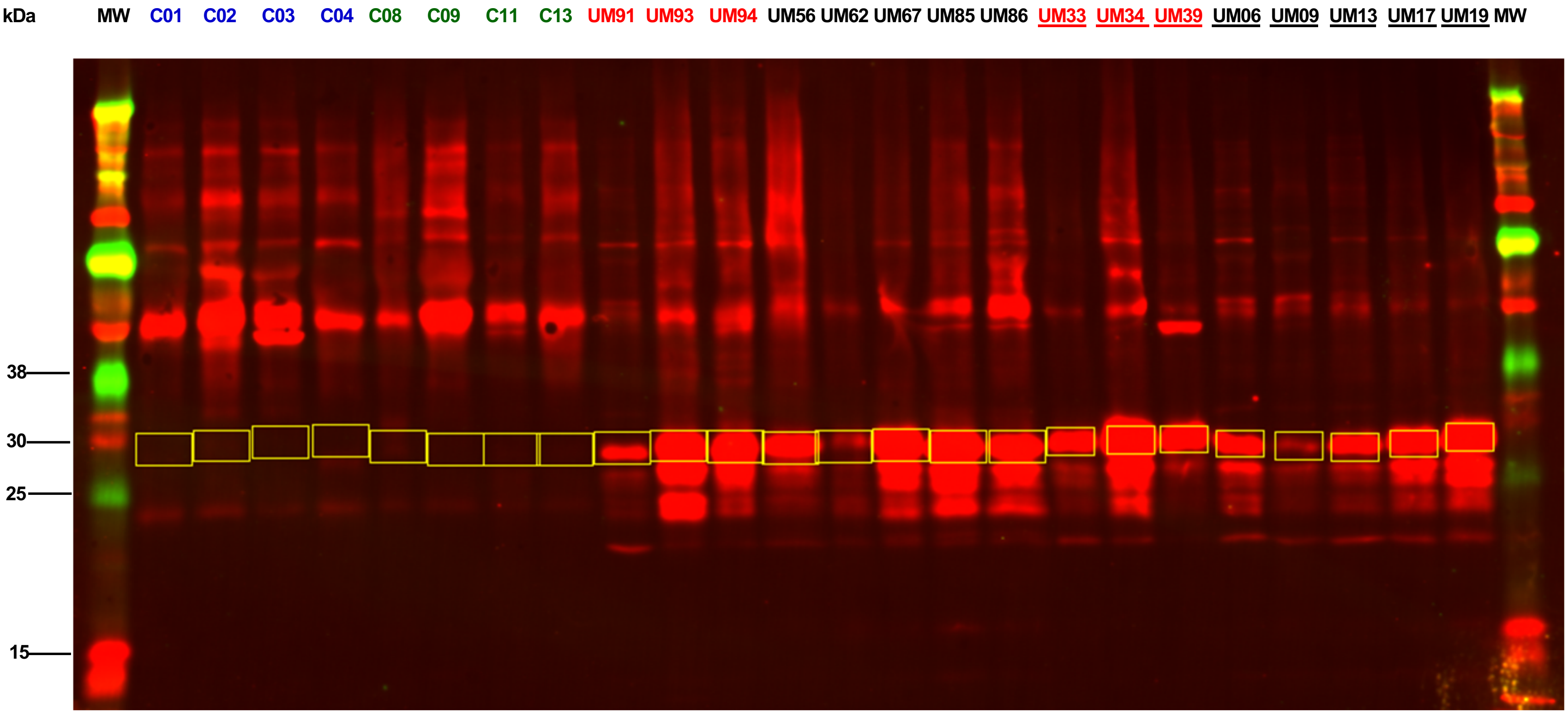


| Figure S3C. Quantitation | | | | | | | | | | | | | | | | | | | | | | | | |
|---|---|---|---|---|---|---|---|---|---|---|---|---|---|---|---|---|---|---|---|---|---|---|---|---|
| Syntenin-1 | C01 | C02 | C03 | C04 | C08 | C09 | C11 | C13 | UM91 | UM93 | UM94 | UM56 | UM62 | UM67 | UM85 | UM86 | UM33 | UM34 | UM39 | UM06 | UM09 | UM13 | UM17 | UM19 |
| Fluorescence intensity | 2290 | 2453 | 2203 | 2246 | 2432 | 2267 | 2290 | 2335 | 6568 | 31723 | 32993 | 15595 | 4992 | 38751 | 57420 | 32464 | 10139 | 111403 | 16330 | 11923 | 3241 | 10293 | 18736 | 42560 |
| Average intensity ± RSD | 2315 ± 4% | | | | | | | | 25763 ± 64% | | | | | | | | 29078 ± 127% | | | | | | | |
| Average pUM intensity / Control ± RSD | | | | | | | | | 11.9 ± 64% iTRAQ=1.6 | | | | | | | | 12.1 ± 127% iTRAQ=1.2 | | | | | | | |
| Ave pUM Met / Ave pUM NoMet | | | | | | | | | 1.0 iTRAQ=1.3 | | | | | | | | | | | | | | | |

***Figure S3D. Fluorescence immunoblot reactivity for G-protein coupled receptor 143, UniProt accession number P51810, molecular weight 43,878 Da.*** The blot was probed overnight at 4°C with anti-G-protein coupled receptor 143 (rabbit pAb at 1:2000, #A10568, ABclonal). Secondary antibody was goat anti-rabbit IgG from LI-COR, USA (#925-6807) used at a dilution of 1:5000, 2-3 h, room temperature in the dark.

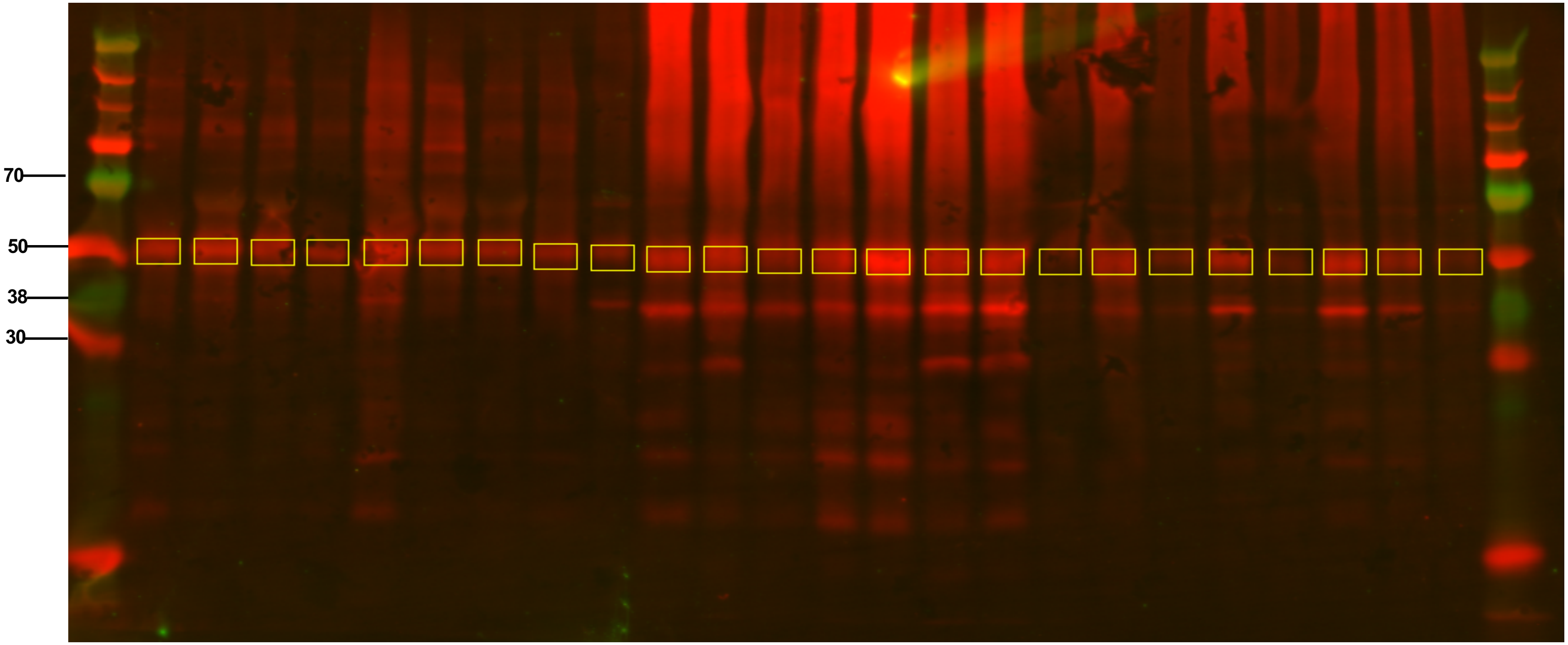


| | | | | | | | | | | | | | | | | | | | | | | | | |
|---|---|---|---|---|---|---|---|---|---|---|---|---|---|---|---|---|---|---|---|---|---|---|---|---|
| **Figure S3D. Quantitation** | | | | | | | | | | | | | | | | | | | | | | | | |
| **G-Potein coupled receptor 143** | **C01** | **C02** | **C03** | **C04** | **C08** | **C09** | **C11** | **C13** | **UM91** | **UM93** | **UM94** | **UM56** | **UM62** | **UM67** | **UM85** | **UM86** | **UM33** | **UM34** | **UM39** | **UM06** | **UM09** | **UM13** | **UM17** | **UM19** |
| **Fluorescence intensity** | 6741 | 8950 | 5907 | 4513 | 9782 | 6903 | 5525 | 4706 | 3642 | 8870 | 9409 | 4957 | 9010 | 26354 | 10412 | 11149 | 2648 | 7116 | 3339 | 6111 | 2904 | 8924 | 5113 | 3031 |
| **Average intensity ± RSD** | 6628 ± 29% | | | | | | | | 10475 ± 66% | | | | | | | | 4898 ± 47% | | | | | | | |
| **Average pUM intensity / Control ± RSD** | | | | | | | | | 1.6 ± 66% iTRAQ=1.9 | | | | | | | | 0.7 ± 47% iTRAQ=1.2 | | | | | | | |
| **Ave pUM Met / Ave pUM NoMet** | | | | | | | | | 2.1 iTRAQ=1.5 | | | | | | | | | | | | | | | |

***Figure S3E. Fluorescence immunoblot reactivity for HLA class II histocompatibility antigen DR alpha, UniProt accession number P01903, molecular weight 28,607 Da.*** The blot was probed overnight at 4°C with anti-HLA class II histocompatibility antigen, DR alpha (rabbit pAb at 1:2000, #A11787, ABclonal). Secondary antibody was goat anti-rabbit IgG from LI-COR, USA (#925-6807) used at a dilution of 1:5000, 2-3 h, room temperature in the dark.

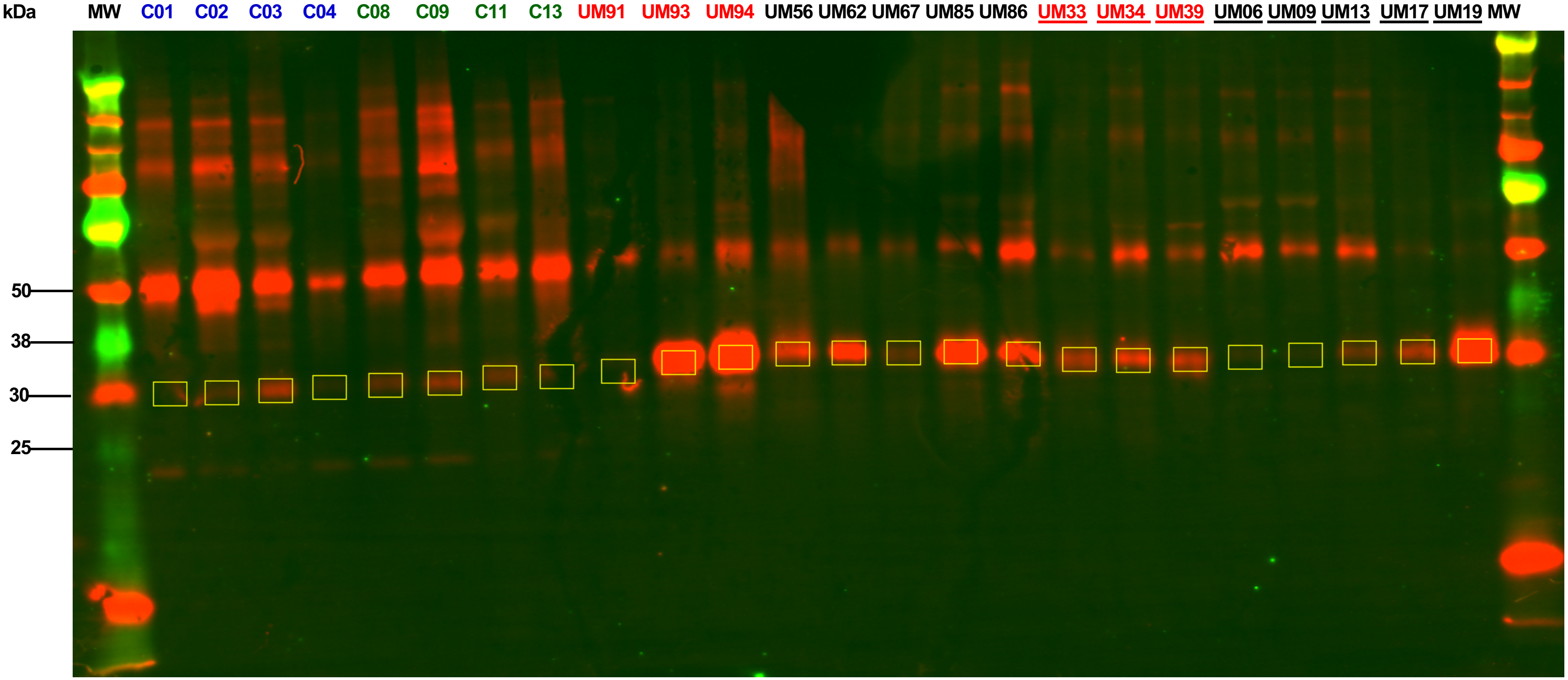


| Figure S3E. Quantitation | | | | | | | | | | | | | | | | | | | | | | | | |
|---|---|---|---|---|---|---|---|---|---|---|---|---|---|---|---|---|---|---|---|---|---|---|---|---|
| HLA Class II histocompatibility antigen DR al | C01 | C02 | C03 | C04 | C08 | C09 | C11 | C13 | UM91 | UM93 | UM94 | UM56 | UM62 | UM67 | UM85 | UM86 | UM33 | UM34 | UM39 | UM06 | UM09 | UM13 | UM17 | UM19 |
| Fluorescence intensity | 1346 | 1487 | 2077 | 1181 | 1545 | 1909 | 1682 | 1204 | 1284 | 10184 | 20579 | 2742 | 3725 | 1633 | 5778 | 3445 | 2317 | 2607 | 2380 | 1252 | 1200 | 1708 | 2219 | 6822 |
| Average intensity ± RSD | 1554 ± 21% | | | | | | | | 6171 ± 105% | | | | | | | | 2563 ± 70% | | | | | | | |
| Average pUM intensity / Control ± RSD | | | | | | | | | 4.09 ± 105% iTRAQ=1.5 | | | | | | | | 1.6 ± 58% iTRAQ=0.9 | | | | | | | |
| Ave pUM Met / Ave pUM NoMet | | | | | | | | | 2.4 iTRAQ=1.7 | | | | | | | | | | | | | | | |

***Figure S3F. Fluorescence immunoblot reactivity for galectin-3 binding protein, UniProt accession number Q08380, molecular weight 65,331 Da.*** The blot was probed overnight at 4°C with anti-galectin-3 binding protein (goat pAb at 0.2 µg/mL, #AF2226, R&D Systems). Secondary antibody was donkey anti-goat from LI-COR, USA (#925-68074) used at a dilution of 1:5000, 2-3 h, room temperature in the dark.

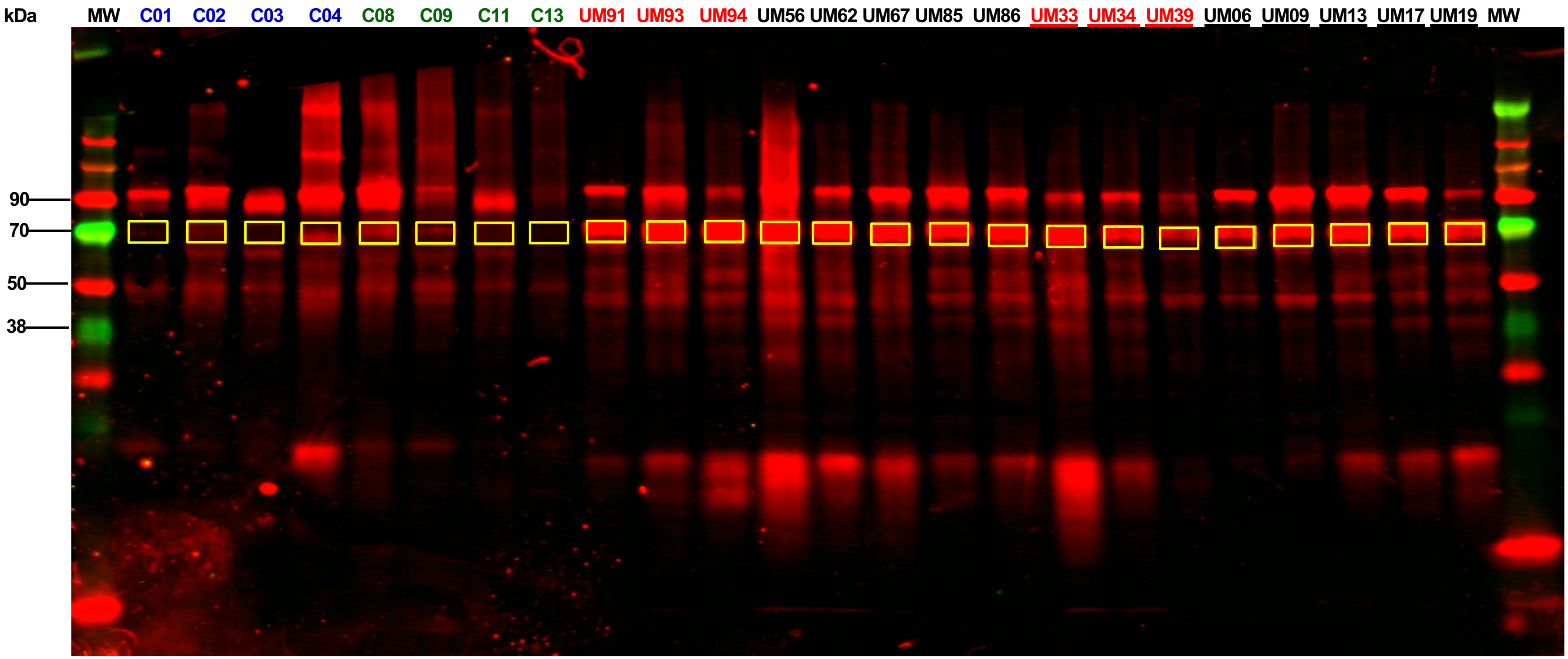


| Figure S3F. Quantitation | | | | | | | | | | | | | | | | | | | | | | |
|---|---|---|---|---|---|---|---|---|---|---|---|---|---|---|---|---|---|---|---|---|---|---|
| Galectin-3 binding protein | C01 | C02 | C03 | C04 | C08 | C09 | C11 | C13 | UM91 | UM93 | UM94 | UM56 | UM62 | UM67 | UM85 | UM86 | UM33 | UM34 | UM39 | UM06 | UM09 | UM13 | UM17 | UM19 |
| Fluorescence intensity | 1721 | 2180 | 1865 | 2832 | 2931 | 2205 | 2030 | 1704 | 6121 | 9236 | 9435 | 13416 | 6324 | 6387 | 6867 | 8142 | 6279 | 4111 | 3145 | 3912 | 4959 | 7837 | 4651 | 4877 |
| Average intensity ± RSD | 2184 ± 22% | | | | | | | | 8241 ± 30% | | | | | | | | 4971 ± 30% | | | | | | | |
| Average pUM intensity / Control ± RSD | | | | | | | | | 3.8 ± 30% iTRAQ=1.1 | | | | | | | | 2.3 ± 30% iTRAQ=0.9 | | | | | | | |
| Ave pUM Met / Ave pUM NoMet | | | | | | | | | 1.7 iTRAQ=1.3 | | | | | | | | | | | | | | | |

***Figure S3G. Fluorescence immunoblot reactivity for vitronectin, UniProt accession number P04004, molecular weight 54,306 Da.*** The blot was probed overnight at 4°C with anti-vitronectin (mouse mAb at 0.4 µg/mL, #MAB2349, R&D Systems). Secondary antibody was donkey anti-mouse from LI-COR, USA (#925-68072) used at a dilution of 1:5000, 2-3 h, room temperature in the dark.

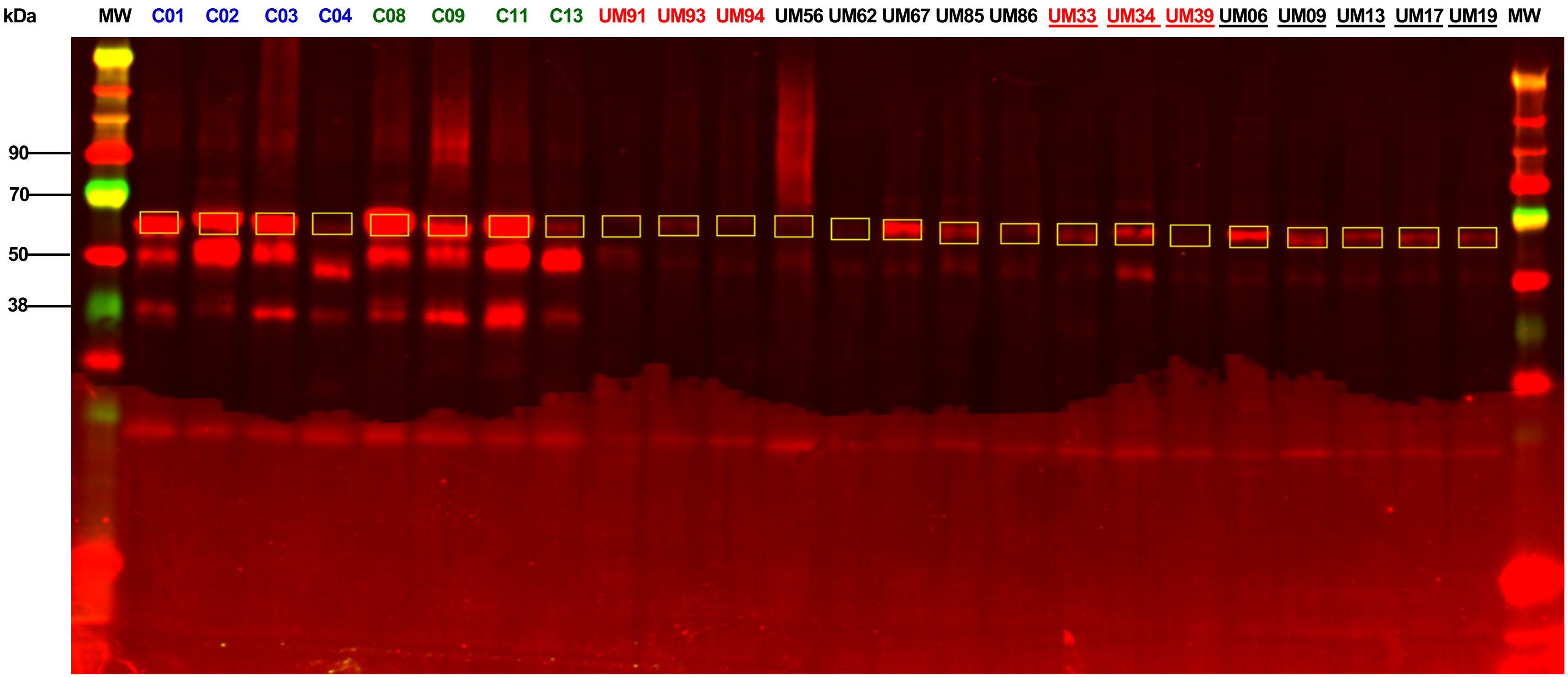


| Figure S3G. Quantitation. | | | | | | | | | | | | | | | | | | | | | | | | |
|---|---|---|---|---|---|---|---|---|---|---|---|---|---|---|---|---|---|---|---|---|---|---|---|---|
| Vitronectin | C01 | C02 | C03 | C04 | C08 | C09 | C11 | C13 | UM91 | UM93 | UM94 | UM56 | UM62 | UM67 | UM85 | UM86 | UM33 | UM34 | UM39 | UM06 | UM09 | UM13 | UM17 | UM19 |
| Fluorescence intensity | 3630 | 5617 | 5906 | 1219 | 4903 | 2597 | 6777 | 1401 | 1103 | 1257 | 1137 | 1230 | 1122 | 2282 | 1565 | 1266 | 1400 | 1661 | 1116 | 2070 | 1595 | 1439 | 1452 | 1351 |
| Average intensity ± RSD | 4006 ± 53% | | | | | | | | 1370 ± 29% | | | | | | | | 1511 ± 18% | | | | | | | |
| Average pUM intensity / Control ± RSD | | | | | | | | | 0.3 ± 29% iTRAQ=0.3 | | | | | | | | 0.4 ± 18% iTRAQ=0.3 | | | | | | | |
| Ave pUM Met / Ave pUM NoMet | | | | | | | | | 0.9 iTRAQ=1.0 | | | | | | | | | | | | | | | |

***Figure S3H. Fluorescence immunoblot reactivity for Nidogen-2, UniProt accession number Q14112, molecular weight 151,254 Da.*** The blot was probed overnight at 4°C with anti-nidogen-2 (goat pAb at 0.3 µg/mL, #AF3385, R&D Systems). Secondary antibody was donkey anti-goat from LI-COR, USA (#925-68074) used at a dilution of 1:5000, 2-3 h, room temperature in the dark.

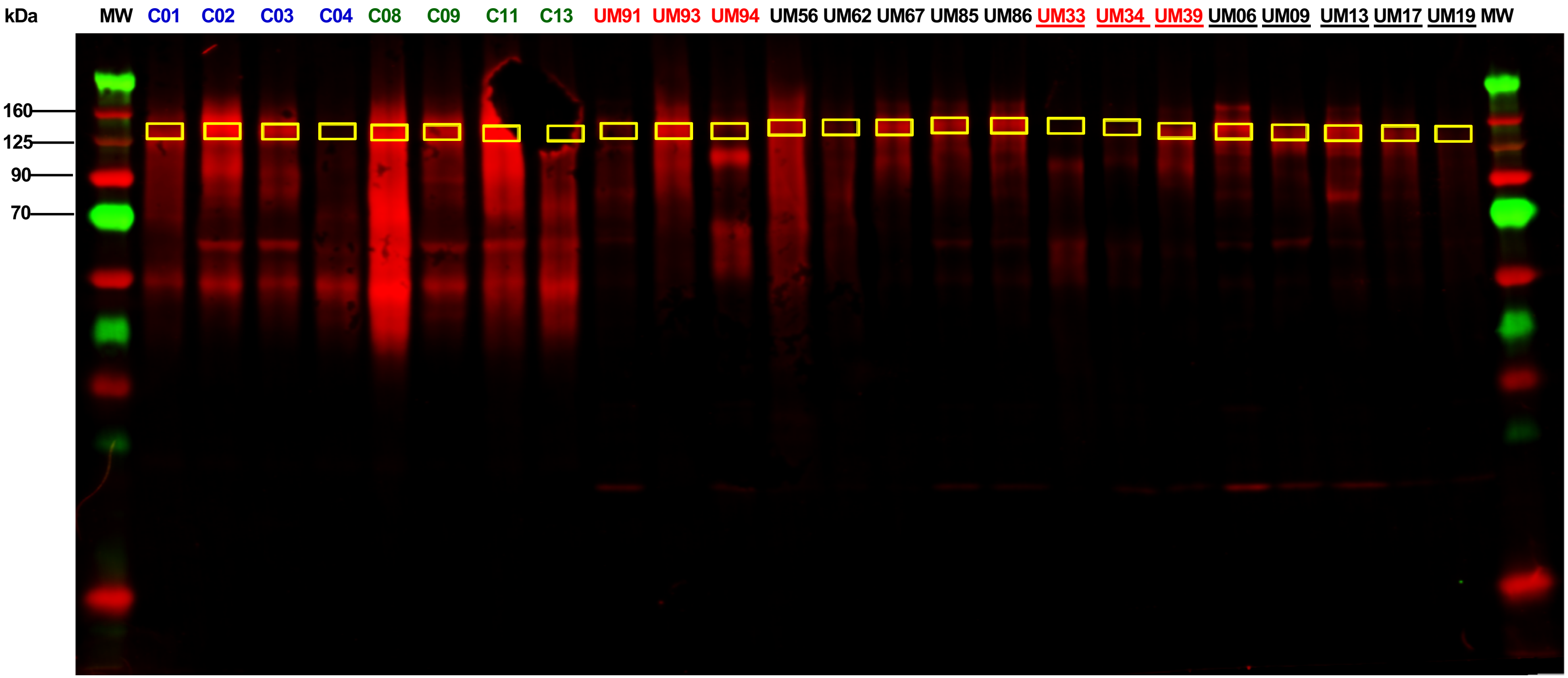


| Figure S3H. Quantitation. | | | | | | | | | | | | | | | | | | | | | | | | |
|---|---|---|---|---|---|---|---|---|---|---|---|---|---|---|---|---|---|---|---|---|---|---|---|---|
| Nidogen-2 | C01 | C02 | C03 | C04 | C08 | C09 | C11 | C13 | UM91 | UM93 | UM94 | UM56 | UM62 | UM67 | UM85 | UM86 | UM33 | UM34 | UM39 | UM06 | UM09 | UM13 | UM17 | UM19 |
| Fluorescence intensity | 3177 | 13715 | 4854 | 991 | 10283 | 4452 | 18177 | 1199 | 1386 | 6303 | 1688 | 4108 | 1424 | 4083 | 3468 | 4277 | 833 | 1140 | 2585 | 5005 | 2544 | 4342 | 2321 | 1041 |
| Average intensity ± RSD | 7106 ± 88% | | | | | | | | 3342 ± 52% | | | | | | | | 2476 ± 62% | | | | | | | |
| Average pUM intensity / Control ± RSD | | | | | | | | | 0.5 ± 52% iTRAQ=0.5 | | | | | | | | 0.3 ± 62% iTRAQ=0.4 | | | | | | | |
| Ave pUM Met / Ave pUM NoMet | | | | | | | | | 1.3 iTRAQ=1.3 | | | | | | | | | | | | | | | |

***Figure S3I. Fluorescence immunoblot reactivity for aspartate aminotransferase, UniProt accession number P00505, molecular weight 47,518 Da.*** The blot was probed overnight at 4°C with anti-aspartate aminotransferase (rabbit pAb at 1:2000, #A6915, ABclonal). Secondary antibody was goat anti-rabbit IgG from LI-COR, USA (#925-6807) used at a dilution of 1:5000, 2-3 h, room temperature in the dark.

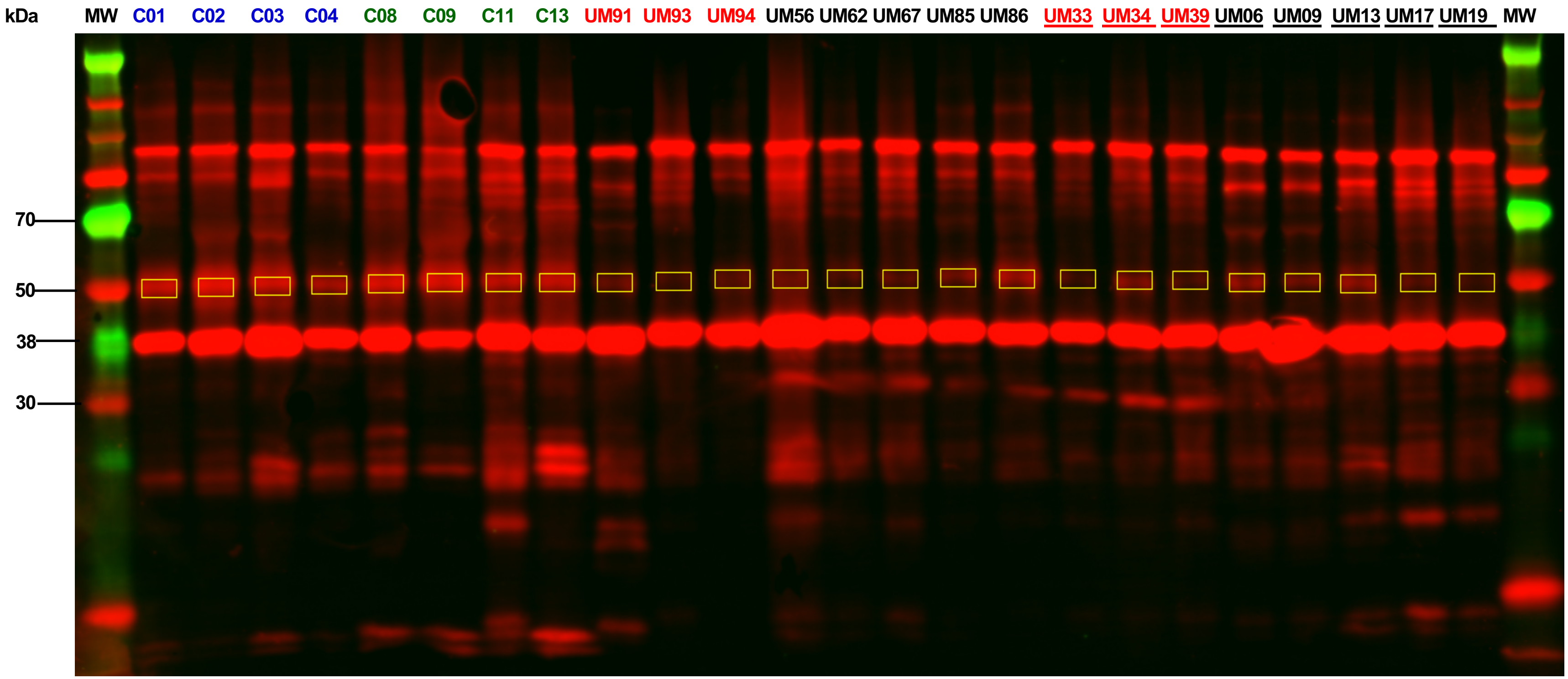


| Figure S3I. Quantitation | | | | | | | | | | | | | | | | | | | | | | | | |
|---|---|---|---|---|---|---|---|---|---|---|---|---|---|---|---|---|---|---|---|---|---|---|---|---|
| Aspartate aminotransferase | C01 | C02 | C03 | C04 | C08 | C09 | C11 | C13 | UM91 | UM93 | UM94 | UM56 | UM62 | UM67 | UM85 | UM86 | UM33 | UM34 | UM39 | UM06 | UM09 | UM13 | UM17 | UM19 |
| Fluorescence intensity | 4333 | 5931 | 4605 | 3059 | 5196 | 5485 | 3992 | 4214 | 1820 | 1394 | 1709 | 2092 | 1490 | 1635 | 2042 | 3080 | 1334 | 2162 | 1484 | 2434 | 1865 | 2554 | 1632 | 1330 |
| Average intensity ± RSD | 4602 ± 20% | | | | | | | | 1908 ± 28% | | | | | | | | 1849 ± 26% | | | | | | | |
| Average pUM intensity / Control ± RSD | | | | | | | | | 0.4 ± 28% iTRAQ=0.5 | | | | | | | | 0.4 ± 26% iTRAQ=0.6 | | | | | | | |
| Ave pUM Met / Ave pUM NoMet | | | | | | | | | 1.0 iTRAQ=0.8 | | | | | | | | | | | | | | | |

***Figure S3J. Fluorescence immunoblot reactivity for lysosome membrane protein 2, UniProt accession number Q14108, molecular weight 54,290 Da.*** The blot was probed overnight at 4°C with anti-lysosome membrane protein 2 (goat pAb at 0.2 µg/mL, #AF1966, R&D Systems). Secondary antibody was donkey anti-goat from LI-COR, USA (#925-68074) used at a dilution of 1:5000, 2-3 h, room temperature in the dark.

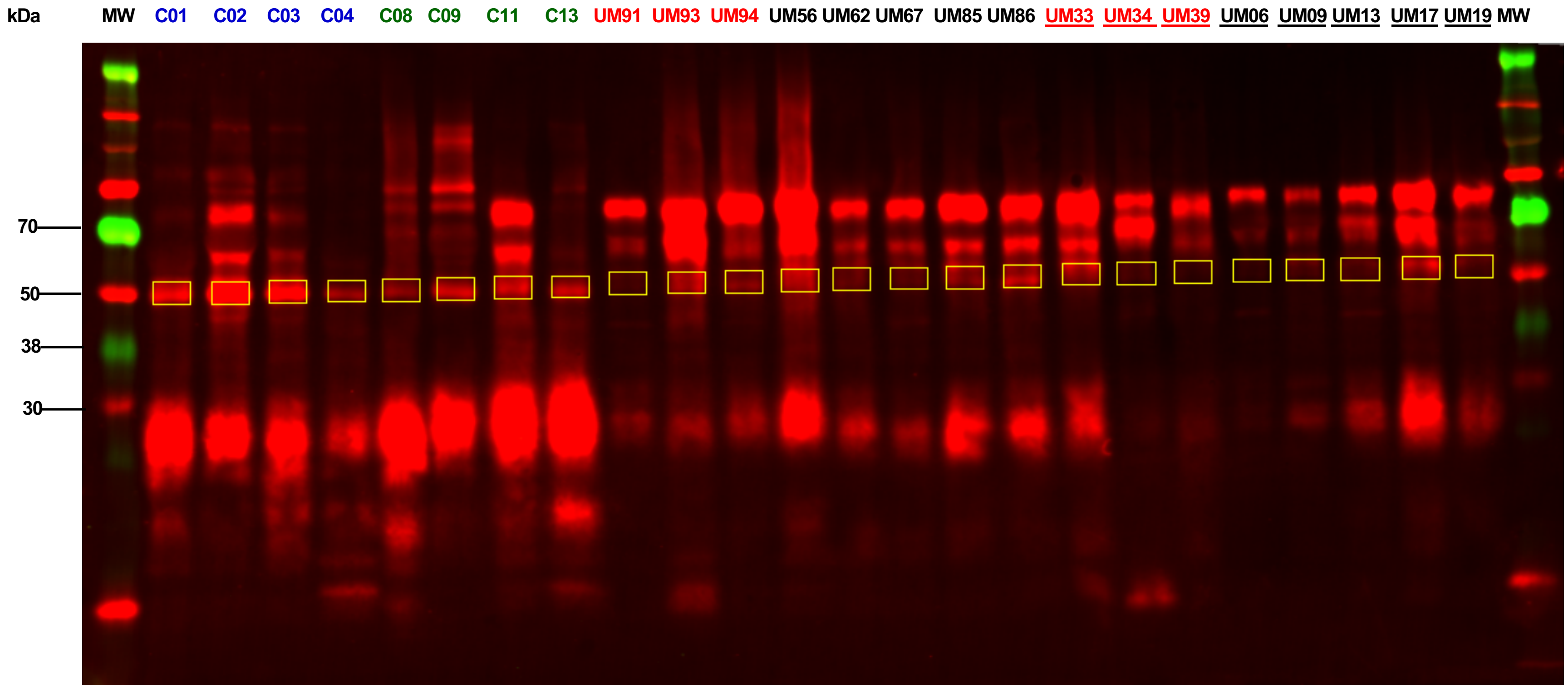


| Figure S3J. Quantitation | | | | | | | | | | | | | | | | | | | | | | | | |
|---|---|---|---|---|---|---|---|---|---|---|---|---|---|---|---|---|---|---|---|---|---|---|---|---|
| Lysosome membrane protein 2 | C01 | C02 | C03 | C04 | C08 | C09 | C11 | C13 | UM91 | UM93 | UM94 | UM56 | UM62 | UM67 | UM85 | UM86 | UM33 | UM34 | UM39 | UM06 | UM09 | UM13 | UM17 | UM19 |
| Fluorescence intensity | 3597 | 5836 | 4252 | 2131 | 2125 | 2886 | 3087 | 2681 | 1431 | 2923 | 2152 | 3044 | 1796 | 1674 | 2418 | 2835 | 2729 | 1419 | 1236 | 1101 | 1496 | 1583 | 2778 | 1569 |
| Average intensity ± RSD | 3324 ± 37% | | | | | | | | 2284 ± 27% | | | | | | | | 1739 ± 37% | | | | | | | |
| Average pUM intensity / Control ± RSD | | | | | | | | | 0.7 ± 27% iTRAQ=0.8 | | | | | | | | 0.5 ± 37% iTRAQ=0.7 | | | | | | | |
| Ave pUM Met / Ave pUM NoMet | | | | | | | | | 1.3 iTRAQ=1.2 | | | | | | | | | | | | | | | |

***Figure S3K. Fluorescence immunoblot reactivity for lactoylglutathione lyase, UniProt accession number Q04760, molecular weight 20,778 Da.*** The blot was probed overnight at 4°C with anti-lactoylglutathione lyase (rabbit pAb at 1:800, #A1932, ABclonal). Secondary antibody was goat anti-rabbit IgG from LI-COR, USA (#925-6807) used at a dilution of 1:5000, 2-3 h, room temperature in the dark.

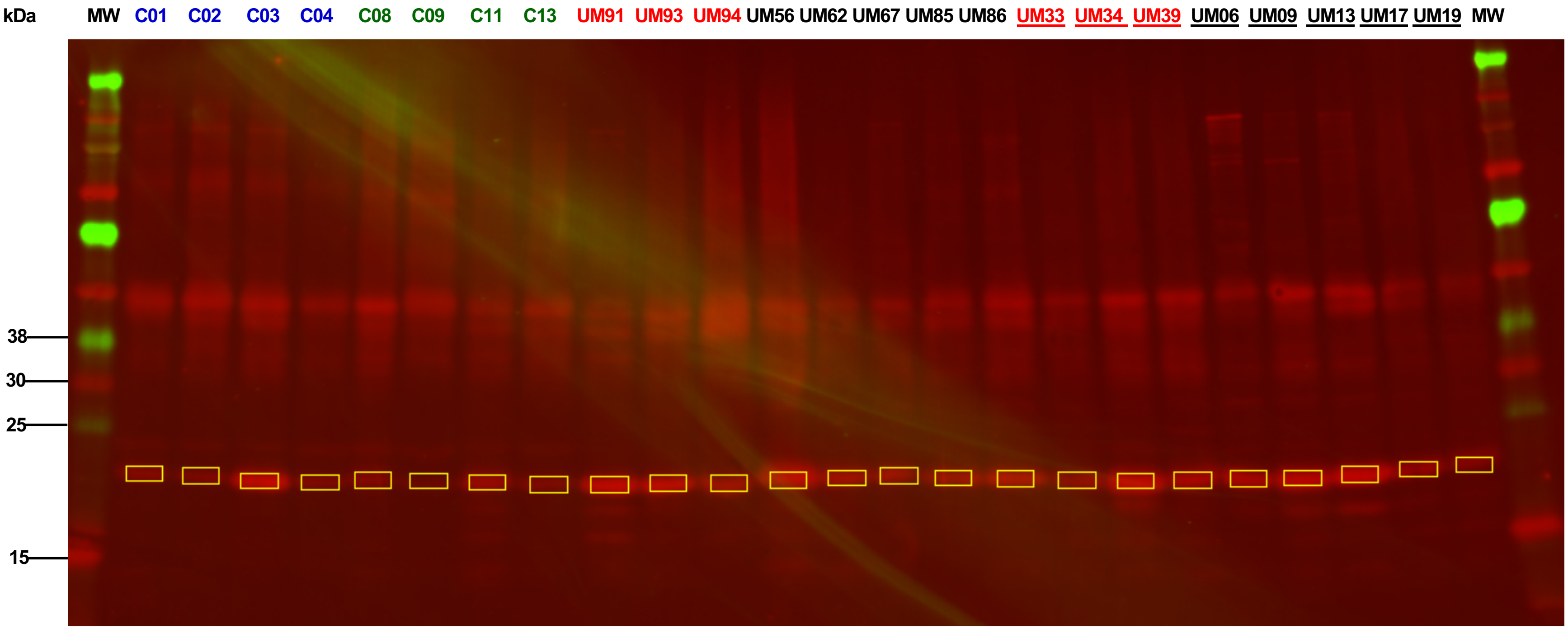


| Figure S3K. Quantitation | | | | | | | | | | | | | | | | | | | | | | | | |
|---|---|---|---|---|---|---|---|---|---|---|---|---|---|---|---|---|---|---|---|---|---|---|---|---|
| Lactoylglutathione lyase | C01 | C02 | C03 | C04 | C08 | C09 | C11 | C13 | UM91 | UM93 | UM94 | UM56 | UM62 | UM67 | UM85 | UM86 | UM33 | UM34 | UM39 | UM06 | UM09 | UM13 | UM17 | UM19 |
| Fluorescence intensity | 1622 | 1230 | 13587 | 1385 | 1645 | 719 | 3378 | 1362 | 12370 | 10417 | 3868 | 11816 | 4280 | 3186 | 3889 | 6563 | 2601 | 14444 | 5689 | 5825 | 10490 | 13348 | 2749 | 1520 |
| Average intensity ± RSD | 3116 ± 138% | | | | | | | | 7049 ± 55% | | | | | | | | 7083 ± 71% | | | | | | | |
| Average pUM intensity / Control ± RSD | | | | | | | | | 2.3 ± 55% iTRAQ=0.8 | | | | | | | | 2.3 ± 71% iTRAQ=1.0 | | | | | | | |
| Ave pUM Met / Ave pUM NoMet | | | | | | | | | 1.0 iTRAQ=0.8 | | | | | | | | | | | | | | | |

***Figure S3L. Fluorescence immunoblot reactivity for cAMP-dependent protein kinase type II-alpha regulatory subunit, UniProt accession number P13861, molecular weight 45,518 Da.*** The blot was probed overnight at 4°C with anti-cAMP-dependent protein kinase type II-alpha regulatory subunit (rabbit mAb at 0.6 µg/mL, # MAB8000, R&D Systems). Secondary antibody was goat anti-rabbit IgG from LI-COR, USA (#925-6807) used at a dilution of 1:5000, 2-3 h, room temperature in the dark.

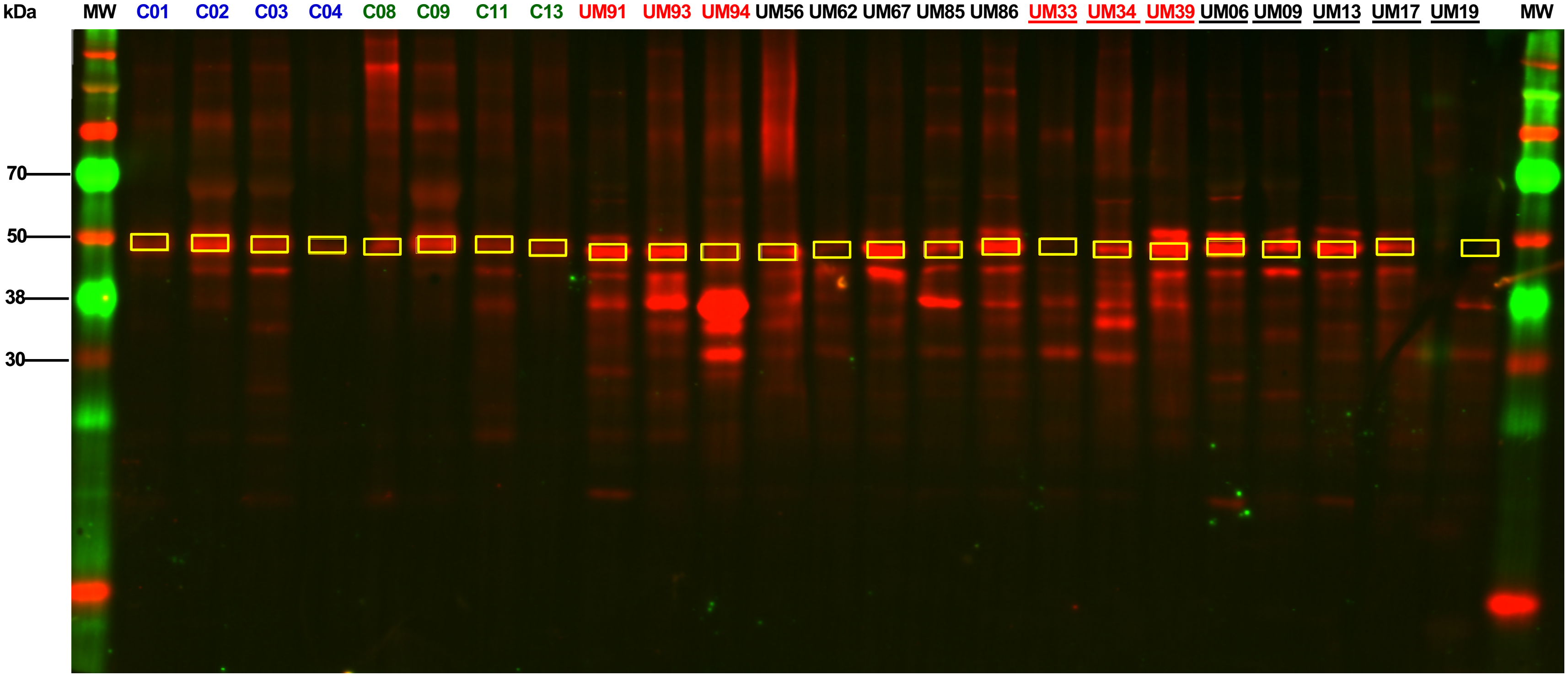


| Figure S3L. Quantitation | | | | | | | | | | | | | | | | | | | | | | | | |
|---|---|---|---|---|---|---|---|---|---|---|---|---|---|---|---|---|---|---|---|---|---|---|---|---|
| cAMP-Dependent protein kinase type II-alp | C01 | C02 | C03 | C04 | C08 | C09 | C11 | C13 | UM91 | UM93 | UM94 | UM56 | UM62 | UM67 | UM85 | UM86 | UM33 | UM34 | UM39 | UM06 | UM09 | UM13 | UM17 | UM19 |
| Fluorescence intensity | 1624 | 2800 | 2095 | 1198 | 1644 | 2506 | 1627 | 1633 | 3401 | 3097 | 2040 | 2239 | 1175 | 4421 | 2074 | 3454 | 890 | 2176 | 4494 | 4094 | 2234 | 2982 | 2009 | 584 |
| Average intensity ± RSD | 1891 ± 28% | | | | | | | | 2738 ± 38% | | | | | | | | 2433 ± 57% | | | | | | | |
| Average pUM intensity / Control ± RSD | | | | | | | | | 1.4 ± 38% iTRAQ=0.6 | | | | | | | | 1.3 ± 57% iTRAQ=0.8 | | | | | | | |
| Ave pUM Met / Ave pUM NoMet | | | | | | | | | 1.1 iTRAQ=0.8 | | | | | | | | | | | | | | | |
